# Review of Non-Invasive Glucose Sensing Techniques: Optical, Electrical and Breath Acetone

**DOI:** 10.3390/s20051251

**Published:** 2020-02-25

**Authors:** Maryamsadat Shokrekhodaei, Stella Quinones

**Affiliations:** 1 Department of Electrical and Computer Engineering, The University of Texas at El Paso, El Paso, TX 79968, USA; 2 Department of Metallurgical, Materials and Biomedical Engineering, The University of Texas at El Paso, El Paso, TX 79968, USA; stellaq@utep.edu

**Keywords:** glucose, non-invasive, diabetes, optical, absorption, scattering, Raman, polarimetry, bioimpedance, breath acetone

## Abstract

Annual deaths in the U.S. attributed to diabetes are expected to increase from 280,210 in 2015 to 385,840 in 2030. The increase in the number of people affected by diabetes has made it one of the major public health challenges around the world. Better management of diabetes has the potential to decrease yearly medical costs and deaths associated with the disease. Non-invasive methods are in high demand to take the place of the traditional finger prick method as they can facilitate continuous glucose monitoring. Research groups have been trying for decades to develop functional commercial non-invasive glucose measurement devices. The challenges associated with non-invasive glucose monitoring are the many factors that contribute to inaccurate readings. We identify and address the experimental and physiological challenges and provide recommendations to pave the way for a systematic pathway to a solution. We have reviewed and categorized non-invasive glucose measurement methods based on: (1) the intrinsic properties of glucose, (2) blood/tissue properties and (3) breath acetone analysis. This approach highlights potential critical commonalities among the challenges that act as barriers to future progress. The focus here is on the pertinent physiological aspects, remaining challenges, recent advancements and the sensors that have reached acceptable clinical accuracy.

## 1. Introduction

Diabetes mellitus is a metabolic disorder in which blood glucose levels exceed 230 mg/dL (known as hyperglycemia) or decrease below 65 mg/dL (known as hypoglycemia) [1]. Patients with diabetes are unable to produce or properly use the hormone insulin. Insulin is a glucose regulatory hormone that interacts with insulin receptors, a process that allows cells to absorb glucose as a source of energy. The number of diabetics in the world is substantial and is increasing. The World Health Organization (WHO) estimates 693 million diabetics (age 18–99 years) worldwide by 2045, compared to 451 million in 2017 [2]. In the US, the number of diabetics is estimated to increase by 54%, from 35.6 million in 2015 to more than 54.9 million by 2030 [3]. This growth is estimated to increase the total annual cost associated with diabetes (medical and non-medical) by 53%, from $407.6 billion in 2015 to more than $622.3 billion by 2030 [3]. Long term diabetes leads to chronic complications such as heart disease, kidney disease, stroke, vision loss and nervous system damage [4].

Diabetes is classified into four groups: type 1 diabetes (T1D), type 2 diabetes (T2D), gestational diabetes due to pregnancy, and other types of diabetes (caused by monogenic diabetes syndromes, diseases of the exocrine pancreas or drug induced diabetes) [5]. In type 1 diabetes, beta (β) cells in the pancreas are destroyed due to an autoimmune response and without β cells to detect glucose, insulin is not released into the bloodstream. Without insulin, cells cannot absorb glucose and thus glucose in the body rises to dangerous levels. As a result, type 1 diabetics need controlled insulin supply to maintain constant blood glucose levels. Type 2 diabetics produce insulin, but the body does not respond properly to the insulin hormone. The insulin receptors that allow glucose to enter cells may be damaged or desensitized to insulin [6]. Type 2 diabetics may have normal or elevated insulin levels; however, this may be insufficient to compensate for the body’s insulin resistance [5]. Type 2 diabetics need to enhance their body’s sensitivity to insulin which can be accomplished by exercise, diet, insulin therapy and weight loss.

Frequent monitoring, ease of blood glucose measurement, real-time measurement and accuracy is instrumental for better control and management of diabetes. Self-monitoring blood glucose (SMBG) by the conventional finger prick method is the most accurate glucose detection method to date. However, this method is painful, inconvenient and carries a risk of infection, especially for patients who are required to check their blood glucose levels several times a day. Commercial blood glucose devices are mostly enzymatic-based electrochemical sensors and involve enzyme-catalyzed reactions. Recently, non-enzymatic-based electrochemical sensors have been developed using multiple nanocomponents and they have exhibited a fast response and high sensitivity [7,8,9,10,11]. Despite significant developments in the evolution of the electrochemical sensors, fully non-invasive glucose monitoring approaches still are in high demand since they have the potential to be reliable, sensitive, user-friendly and result in tailored treatment options.

Clarke error grid analysis is a common method frequently used to quantify the accuracy of measured glucose values compared to reference glucose measurements [12]. The Clarke error grid is divided into five zones (A, B, C, D and E), that depend on the relationship between the measured and reference glucose values [13]. The data values that fall in zone A mean that the measured glucose values are within 20% of the reference values and the recommended treatment based on this level of error is still appropriate for the patient. The same is true for zone B, although the data values do not fall within 20% of the reference values. Data falling into zone C includes enough of an error that the associated treatment would be unnecessary for the patient. If the error is sufficient to land in zone D, hyperglycemia or hypoglycemia would not be properly diagnosed. Zone E data would recommend treatment that is inappropriate for either hypoglycemic or hyperglycemic patients [13]. Clarke error grid has discontinuous transitions between zones. This means that any small change in measured glucose value can move the result from a zone with ideal clinical accuracy to another zone with non-optimal clinical accuracy. In this case, the recommended treatment would not the optimal one. Also, the Clarke error grid does not differentiate between different types of diabetes. To compensate for these limitations, the Parkes or consensus error grid (PEG) and the surveillance error grid (SEG) analysis were developed. The Parkes or consensus error grid includes five zones (A to E) with a continuous transition between zones and includes one grid for T1D patients and a second grid for T2D patients. The surveillance error grid includes zones with different colors from green to red. Data pairs falling into the red zone include the highest level of error compared to zones with different colors [14].

Tura et al., Poddar et al., Uwadaira and Ikehata, Oliver et al., and Gonzales et al. collectively provide extensive overviews associated with invasive, minimally invasive and non-invasive glucose measurement techniques [15,16,17,18,19]. Bruen et al., Kim et al., and Vashist reviewed glucose measurement techniques based on physiological fluids such as interstitial fluid, urine, sweat and salvia [20,21,22]. Koschinsky and Heinemann described the critical clinical and technical factors of minimally invasive and non-invasive glucose sensors [23]. Lin et al. highlighted the satisfactory function for in-home use of eight past and current non-invasive monitoring devices [24].

This review paper aims to add to the literature by identifying and summarizing interdisciplinary fundamental information that connects the sensor functionality to the importance of several physiological factors associated with non-invasive glucose sensing. Non-invasive blood glucose measurement approaches are categorized based on their reliance on: (1) glucose properties, (2) tissue properties and (3) the acetone level in exhaled breath. For each of these categories, the challenges and limitations affecting glucose readings are identified, and the recent advances in non-invasive glucose sensing are identified from each category to address associated challenges.

**Figure 1:**
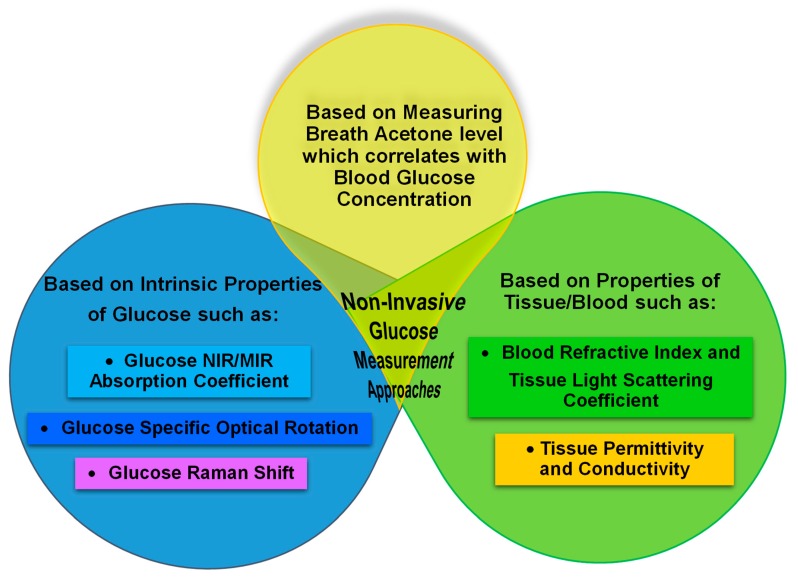
Non-invasive glucose sensing techniques.

**Figure 2:**
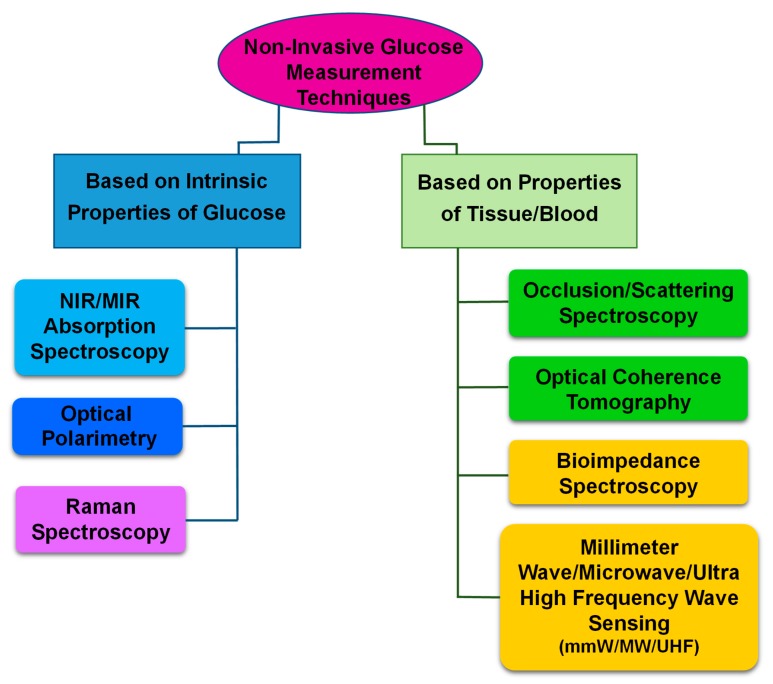
Non-Invasive glucose measurement techniques based on glucose and tissue/blood properties.

Figure 1 illustrates three categories of non-invasive glucose measurement approaches based on measuring intrinsic properties of the glucose, properties of tissue and breath acetone measurements that relate to glucose concentration. The glucose absorption coefficient, glucose specific optical rotation and glucose Raman shift are intrinsic properties of glucose. The tissue light scattering coefficient, tissue permittivity and tissue conductivity are tissue properties that can be used to measure glucose concentration. Exhaled breath studies focus on the measurement of blood glucose through breath analysis, including levels of acetone. The measurement techniques associated with the two first categories are listed in Figure 2. For the third category, 17 experiments (14 articles) that have attempted to measure blood glucose via measuring breath acetone are closely reviewed and summarized in terms of experimental conditions. The exhaled breath studies highlight the various parameters that result in conflicting in glucose concentrations measurement using this technique.

Section 2 is a brief overview of the physiological aspects related to glucose distribution in the body, skin and tissue layers. Ketogenesis is discussed along with the production of ketones (such as acetone), and how it correlates with blood glucose concentration. This concise background provides the reader with the fundamental physiological knowledge and related functional factors that should be considered when designing non-invasive glucose measurement devices. Section 3, Section 4 and Section 5 describe the fundamental concepts associated with glucose measurements based on intrinsic glucose properties, optical/electrical characteristics of tissue, and breath acetone studies, respectively. Each section includes the underlying challenges associated with these techniques along with possible approaches to overcome these obstacles. Sensors under development are identified for each category along with their level of accuracy. Finally, Section 6 provides critical thinking and discusses possible improvements that can be made for further development.

## 2. Background

The more we know about how glucose molecules interact with blood/tissue components, the better we solve issues associated with glucose sensing. The background about glucose transportation in the body, skin tissue layers in terms of thickness and composition is presented here which is necessary to know before elaborating on non-invasive glucose measurement techniques (in Section 3 and Section 4). Further physiological concepts that are related to glucose sensing will explain concerning each method separately at the rest of the paper. Physiological concepts related to the correlation of blood glucose level and breath acetone level are described here by presenting details about the ketogenesis process and production of ketones in diabetes.

### 2.1. Glucose Transport in the Body

The total blood volume in the human body consists of approximately 60% plasma, 40% erythrocytes (red blood cells) and less than 1% buffy coat (leukocytes/white blood cells and thrombocytes/platelets) [25]. Plasma is primarily made up of water (~90%), and includes approximately 7% proteins, 0.5% inorganic salts, 0.4–0.7% lipids, 0.07–0.1% glucose, and less than 0.07% lactic acid, carbamide and amino acids [26]. The glucose in plasma is carried in blood arteries and is transported through the circulatory system through arterioles to capillaries. The level of glucose in arterial blood and capillary blood is found to be almost identical [27]. Once in the capillaries, glucose diffuses into the interstitial fluid which surrounds tissue cells. Glucose is converted to energy and it is used or stored for later consumption. There is a time lag between glucose levels in capillary blood versus the glucose diffused to the interstitial fluid. The blood from capillaries flows back to the heart through small vessels or venules which is then transported through larger veins back to the heart. As a result of this process (supply of glucose to tissue), the glucose level in the arterial blood is higher than the glucose traveling back through the veins at any given time [27].

The traditional glucose monitoring finger prick method that collects glucose samples collectively from the dermis layer and capillaries also captures a small sample portion from arterioles and venules. As mentioned before, the level of glucose within the arteries and capillaries (in blood plasma) is not the same as the level of glucose in the interstitial fluid at any given time. There is a delay of approximately 5 to 15 min with respect to the amount of glucose in interstitial fluid compared to the glucose values in arteries and capillaries [28]. Any glucose changes measured within the tissue (i.e., coming from the interstitial fluid) will not represent a concurrent change of glucose level in the blood due to this delay. This delay is not constant, may differ between individuals and depends on the blood flow, permeability of the capillary, glucose concentration gradients (there is a higher lag time during a rapid change in blood glucose concentration), the rate of glucose uptake (which itself depends on insulin level), etc. [29,30].

It is important to be aware of these physiological effects since non-invasive methods may detect glucose within the interstitial fluid and these measurements are not reflective of glucose trends in blood plasma in real-time. Shi et al. developed a theoretical model that describes the correlation between interstitial fluid and blood glucose concentration based on the Starling equation and Fick’s laws (describing the process of glucose diffusion from capillaries into the interstitial fluid) [29]. The developed model includes the effect of physiological factors such as blood flow and glucose permeability and is used to accurately simulate glucose moving time course through capillaries into the interstitial fluid. A possible solution to compensate for the delay associated with tissue glucose measurements is training an artificial neural network model that predicts glucose level ahead of time based on previously recorded glucose concentrations [31]. This approach resulted in 90% clinical accuracy based on error grid analysis.

### 2.2. Skin Tissue Layers

**Figure 3:**
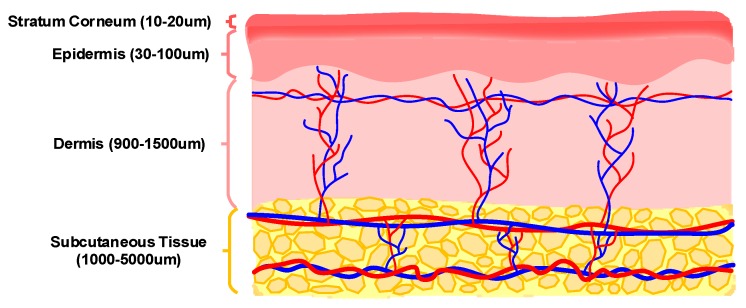
Skin tissue layers.

The skin tissue contains arterioles, venules, capillaries and interstitial fluid, and interstitial fluid occupies significantly more volume compared to blood plasma. As illustrated in Figure 3, skin tissue layers include the stratum corneum (10–20 µm), the epidermis (30–100 µm), the dermis (900–1500 µm) and the subcutaneous tissue (1000–5000 µm) [32]. The epidermis layer includes approximately 15–35% interstitial fluid and no blood vessels. The dermis layer contains arterioles, venules and capillaries and approximately 40% interstitial fluid. The subcutaneous tissue includes fat storage, some interstitial fluid (less than in the dermis layer) and blood vessels connecting the dermis to the blood circulating in the body [27,33,34,35]. Each skin layer has its own optical and dielectric characteristics, which may vary between individuals due to differences in morphology and thickness of skin layers, the concentration of tissue/blood components (such as glucose), cutaneous blood perfusion, etc. The most abundant cells in the blood are red blood cells (RBCs) with biconcave disc shape and have a significant effect on the dielectric property of tissue. RBCs change their shape by rearranging their cytoskeleton during changes in glucose concentration. This conformational variation causes a change in the dielectric property of red blood cells. In vivo measurements demonstrated an increase in the dielectric permittivity of RBCs due to a decrease in glucose concentration [36]. Differences in size, morphology and distribution of RBCs between individuals result in differences in dielectric properties of tissue regardless of glucose concentration, and so affect the accuracy of glucose reading using dielectric based glucose measurement techniques.

### 2.3. Glucose Storage in the Body 

The hormone insulin controls blood glucose levels and helps cells to take in glucose as a source of energy. If the body has sufficient energy, “insulin hormone stimulates the liver to store glucose as glycogen” via a process called glycogenesis [37]. Glycogen storage in the muscles and liver is limited, so if there is already enough glycogen in the muscles and liver, the body converts excess glucose into fatty acids and stores them as triglycerides via a process called lipogenesis [38]. In contrast to limited glycogen stores, body fat stores are virtually unlimited. Fat stores provide humans with enough energy to sustain them for long periods without enough food.

### 2.4. Ketogenesis and Production of Ketones

The human body can reach insufficient available glucose and stored glycogen levels overnight when a person is sleeping, during low carbohydrate dieting, low food intake (fasting) and intense exercise [39]. At this time, the body starts to break down fatty acids into high-energy compounds called ketones. This process is called ketogenesis and is a normal process that occurs in all healthy human bodies. Ketogenesis is beneficial for people who intend to burn fat for fuel in order to lose weight. Three types of ketone bodies are: (1) acetoacetate, (2) β-hydroxybutyrate, and (3) acetone [39]. Muscle and other tissue pick up ketone units to supply energy to the human body. The rate of ketogenesis depends upon the activity of inhibitor enzymes and stimulator enzymes. The enzymes controlled by insulin, such as lipase and acetyl CoA carboxylase, inhibit ketogenesis. Epinephrine and glucagon are hormones that stimulate ketogenesis [40].

### 2.5. Influence of Diabetes on Ketogenesis Process

In a healthy individual, the insulin and glucagon balance the level of ketones, and this controls the rate of ketogenesis. However, this scenario is very different for people with diabetes who suffer from insufficient levels of insulin. Insulin acts as an inhibitor for ketone production, and a lack of insulin leads to high levels of ketones. As a result, the body undergoes a pathological process called diabetic ketoacidosis (DKA) [40]. A high level of ketones in the blood is observed in patients with Type 1 diabetes who are insulin dependent. DKA is seldom present in patients with Type 2 diabetes, with the exception of ethnic minorities [5]. An increase in ketone concentration due to lack of insulin in Type 1 diabetic patients is accompanied by a decrease in plasma pH. Very high and dangerous levels of blood ketones can decrease plasma pH to low levels, and this can lead to coma or death if not reversed [41].

A fraction of ketones not used by the body (excess ketones) spill over into the urine. One type of ketone, acetone, is produced by the spontaneous decarboxylation of acetoacetate. Breath analysis is a method used to detect acetone since acetone leaves the body via the lungs. The level of acetone is high in diabetic patients compared to healthy individuals [40,42,43,44,45,46] and is thus a possible biomarker for diabetes diagnosis. There are many attempts to measure the exhaled acetone and to correlate it to blood glucose concentration.

## 3. Non-Invasive Glucose Sensing Methods Based on Intrinsic Properties of Glucose

There are several non-invasive glucose sensing techniques that rely on the intrinsic properties of glucose. These include near-infrared/mid-infrared (NIR/MIR) absorption spectroscopy, optical polarimetry and Raman spectroscopy. The intrinsic properties central to each of these measurements are the glucose optical absorption coefficient, specific optical rotation and Raman shift, respectively. Sensors that detect the interaction of light with glucose rely on changes in the NIR/MIR absorption, the rotation angle of light, and the Raman signal intensity.

An optical glucose sensor is comprised of light source(s), a detector, and an optical transducer that converts the detected light into a measurable electrical signal. There are two modes of operation for an optical sensor, reflection and transmission. For reflection mode, both the light source and the photodetector are located on the same side. In transmission mode, the photodetector is located on one side of the sample and the light source is on the opposite side.

**Figure 4:**
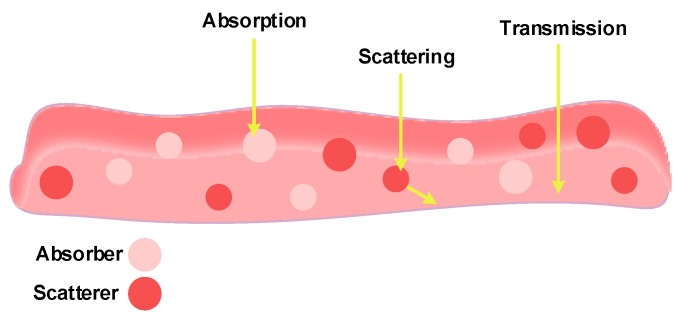
Types of Interactions between Light (Photons) and Tissue.

Strategic glucose sensor locations on the human body include the fingers, ears, lip, forearm, anterior chamber of the eye and across the tongue. As light enters the body, it interacts with atoms within the tissue, and is absorbed, transmitted or scattered, as illustrated in Figure 4. The type of interaction depends on: (1) the wavelength of the incident light, (2) tissue structure, and (3) tissue optical properties (such as relative refractive index, absorption coefficient, and scattering coefficient) [47].

When light is absorbed by a material, the energy associated with the light or photon is used up as a result of the interaction between the light and the material. Depending on the energy of the photon, there can be different types of energy transitions in molecules, including energy transition between vibrational states, rotational states, electronic states, etc.

Depending on the wavelength of incident light (λ1), scattering can be dominant. Elastic and inelastic scattering are two types of scattering of light. For elastic scattering, the energy of scattered light is equal to the energy of incident light (i.e., λ1 = λ_scattered_light_) while in inelastic scattering, the energy of scattered light is less or greater than incident light (i.e., λ1 ≠ λ_scattered_light_) [48,49]. Scattered light can be in any direction/angle (from backscattering to forward scattering), and is affected by material structure and energy of incident light. The light which interacts with tissue components tends to scatter in the forward direction for single scattering. However, multiple scattering may result in backward scattering in tissue.

Elastic scattering includes both Rayleigh scattering and Mie scattering. In Rayleigh scattering, the size of the particles involved in scatterings, such as atoms or molecules, is much less than the wavelength of incident light (λ1). In contrast, for Mie scattering, the size of the particles involved in scattering is comparable to the wavelength of the incident light [47,49]. Rayleigh scattering depends more strongly on the wavelength of the incident light compared to Mie scattering, and the intensity of scattered light in Rayleigh scattering is proportional to (1/$\lambda_{1}^{4}$) [50]. Inelastic scattering including Raman scattering and fluorescence. In this case, the emitted light has a different wavelength than the incident light. As the portion of inelastic scattering is negligible against elastic scattering [48], in optical methods based on inelastic scattering measurement, there would be a highly sensitive detector to detect weak inelastic scattered light and algorithms for improving the signal to noise ratio.

When a material is transparent to light, the photon passes through the material without interacting with the material and thus retains its original energy.

### 3.1. Mid-Infrared and Near-Infrared Spectroscopy

**Figure 5:**
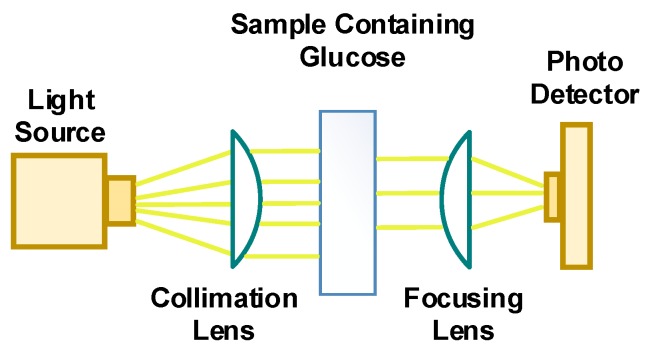
A simplified schematic illustrating transmission absorption spectroscopy.

Mid-infrared (MIR) and near-infrared (NIR) absorption spectroscopy are measurement techniques used to acquire quantitative information about a tissue sample and probe its components. The wavelength range for NIR is between 700 and 2500 nm and for MIR, the range is between 2500 and 25,000 nm. NIR sensing and measurements are achievable in both reflection and transmission modes due to the associated penetration depths of 0.5 mm or more. Whereas, MIR based sensing and measurements methods can only operate in the reflection mode since MIR light cannot penetrate more than a few micrometers through the tissue [18,51,52]. The spectroscopy setup for MIR and NIR absorption includes a light source generating different wavelengths of light in the range of MIR or NIR and a photodetector to measure the intensity of light, which is either reflected or transmitted through the sample depending on the wavelength of light. Figure 5 includes a simplified diagram illustrating the absorption of light through a sample consisting of a mixture of glucose and distilled water solution.

The absorption spectroscopy concept can be understood based on the Beer-Lambert law of absorption (Equation (1)) [16,53]:

(1) $$I=I_{0}10^{\left(-l.\varepsilon .c\right)}=I_{0}e^{\left(-l.\mu_{a}\right)},$$

where *I*_0_ is the initial light intensity (W/cm^2^), *I* is the intensity of light at any depth within the absorption medium in W/cm^2^, *l* is the absorption depth within the medium in cm, *ε* is the molar extinction coefficient or molar attenuation coefficient in L/(mmol cm), which depends on the wavelength of incident light and the structure of the absorbing molecules, and *c* is the concentration of absorbing molecules in mmol/L. The product of *ε* and *c* is proportional to the absorption coefficient (*μ_a_*).

This model assumes that the attenuation of light due to scattering is negligible in comparison to the light being absorbed. The intensity of light that is transmitted/reflected and measured by the photodetector is a function of the concentration of the absorbing molecules, the thickness of the sample, and the absorption coefficient of the absorbing molecules.

Absorbance is defined as log (I_0_/I). Different materials exhibit an absorbance peak within a specific wavelength range due to the dependency of ε on the wavelength of the incident light and the structure of the absorbing molecules. For NIR range, the value of ε for glucose varies between 0 and 1 dL/(g cm) [54]. In Figure 5, the absorbing molecules are distilled water and glucose. In this case, MIR/NIR absorption spectroscopy can measure the variation of glucose absorbance as a function of wavelength and identify the wavelength of light for which the highest glucose absorption occurs.

A spectrometer is a device that measures absorbance vs. a wide range of continuous wavelengths. A lensed optical fibers probe can be used to interface between the body and a spectrometer to take body measurements [55]. The design of an absorption-based device for self-glucose monitoring should be portable as well as small enough for in vivo applications. It is evident that blood glucose sensing is more complicated than measuring the dissolved glucose in a sample of distilled water and glucose solution. There are several critical issues that need to be considered and resolved when using a MIR or NIR based absorption method in sensing blood glucose. Some of these issues and possible solutions are described below.

#### 3.1.1. Absorption of Light by Water

The most abundant molecules in biological fluids in the human body are water while glucose comprises only 0.07–0.1% of the blood plasma [26]. Water molecules absorb a significant percentage of the incident light, especially within the range of MIR wavelengths. When this occurs, the absorption of light by the water is independent and in addition to the absorption of light by the glucose molecules and therefore, decreases the sensitivity to glucose molecules. So, it is necessary to identify a wavelength window that minimizes the absorption of light by water and maximizes the absorption of light by the glucose molecules.

There are two absorption peaks for water in the NIR range: one is placed between 1350 and 1520 nm, and the other is between 1790 and 2000 nm [56]. The NIR wavelength window between 700 and 1100 nm, between 1500 and 1850 nm and between 2000 and 2400 nm are used for glucose measurement since glucose has observable absorption and relatively minimal amount of light absorption by water occurs [57,58,59,60]. Light absorption by glucose is higher in the wavelength range between 2000 and 2400 nm compared to the shorter ranges (1500–1850 nm and 700–1100 nm), while light absorption by water is lower for the shorter ranges. So, using shorter wavelengths can result in higher selectivity to glucose molecules by minimizing the interfering effect of water [61].

Absorbance spectra for glucose in MIR range between 6250 and 11,110 nm (900–1600 cm^−1^) indicate that glucose has several absorption peaks which are placed between 8696 and 10,000 nm (1000–1150 cm^−1^) [6]. Absorption of light by water is significant in the MIR range compared to the NIR range. A possible solution to minimize the interfering effect of water is using multiple wavelengths [62,63]. Guo et al. used two discrete MIR wavelengths at 9500 nm and 10,400 nm, where the 9500 nm light is absorbed by both glucose and water, and the 10,400 nm is absorbed mostly by water [62]. The differential method was applied in order to subtract the absorption of water, resulting in a signal that mostly represents the absorption of glucose. 

The multiple wavelength approach was applied in a non-invasive glucose measurement device called TensorTip Combo Glucometer. TensorTip Combo Glucometer was designed by Cnoga Medical Ltd (Caesarea, Israel) and the concept approval process started in 2006. The device was approved for use in numerous countries worldwide. TensorTip Combo Glucometer is capable to measure glucose in the range between 70 and 440 mg/dL [64]. The device is comprised of four LEDs with wavelengths between 600 and 1000 nm and a color image sensor camera that photographs the transmitted light which passes through the fingertip. Glucose reading is based on the analysis of six-dimensional signal (position [x, y], time [t], color [red green blue]) which correlates with blood glucose concentration. The performance of the sensor was investigated in [65,66] by conducting a study on 14 healthy subjects, six T1D patients and 16 T2D patients. Based on the consensus error grid, 100% of data were demonstrated to be in zone A (96.6%) and B (3.4%) [65].

#### 3.1.2. Absorption of Light by Blood Components and Tissue

The concentration of the absorbing molecules in a solution is measured based on the change in the light intensity as it passes through the solution according to Equation (1). This equation predicts the absorption of light by a mixture of glucose in distilled water. However, in reality, the effect of other blood components and absorbing tissue components affect the amount of light absorbed. As a result, the absorption coefficient is the summation of the absorption coefficients of all the absorbing components [47]. The absorbing tissue components include melanin (gives hair and skin their color), beta-carotene (responsible for the yellow color of tissues) and fatty tissues (which vary in different individuals). The absorbing blood components are albumin (3.5–5 g/dL), globulin (2.5–3 g/dL) and hemoglobin (11.5–13.7 g/dL) which are much higher in concentration compared to glucose (0.065–0.105 g/dL), and so their contribution on light absorption can be significant [52,54,67,68]. These components act as interferers while measuring blood glucose. To minimize the absorption due to all unwanted components, the wavelength of the light source should be chosen so that the light source is highly absorbed by glucose and is mostly transparent to blood and tissue components.

Kasahara et al. measured absorption of 1% glucose solution in MIR range between 900 and 1200 cm^−1^, and observed three absorption peaks at 1036, 1080 and 1110 cm^−1^ [69]. They demonstrated glucose measurements within tissue fluid of human oral mucosa using a combination of three different wavenumbers (1050, 1070 and 1100 cm^−1^) at which interfering components such as carbon hydrate residues attached to collagen, phospholipids and nucleic acids in the saliva and mucosa have relatively low absorption [69]. In this research, a delay of 20 mins was observed between change in blood glucose level and the respected change in tissue fluid of oral mucosa.

Maruo and Yamada measured absorption of glucose, protein, fat and water in NIR range between 1300 and 1900 nm, and observed absorption peaks at 1600, 1510, 1727 and 1450 nm, respectively [58]. NIR absorption spectra were measured from human skin at the four wavelengths mentioned above and at 1650 where there is relatively lower light absorption by confounding factors (water, protein and fat). Glucose quantity information was then successfully extracted by formulating a linear combination of absorption data set at 1600, 1510, 1727, 1450 and 1650 nm [58].

MIR absorption by glucose samples and other biological components produces clear and distinct signals [52], while NIR absorption of these samples also includes several overlapping peaks generated by hydrogen-bonded (N-H, C-H, O-H) molecules [6,16,52]. A solution to suppress the effect of these other tissue/blood factors is by using multiple wavelengths instead of a single wavelength as the light source [58]. Glucose quantitative data can be derived from absorbance vs. wavelength data by applying the following analytical and calibration methods: multiple wavelength linear regression [69], partial least squares [54,70], principal component regression, Deming regression [71], support vector machines [72] and so on. These algorithms help to generate a model that accurately predicts the glucose concentration by considering the effect of multiple variables on the output. More specifically, the algorithms weigh the effect of multiple variables by minimizing and or eliminating the influence of competing signals on the real value of the glucose concentration. Finally, the effectiveness of the algorithms can be determined by using evaluation criteria, and comparing the estimated glucose concentration with the known value of glucose concentration [52]. 

#### 3.1.3. Scattering of Light by Blood Components and Tissue

The scattering of light due to multiple tissue components results in deviation from Beer-Lambert law of absorption (Equation (1)), and results in measurement error. In fact, both tissue and blood components cause light scattering, which attenuates the intensity of the measured light. Total attenuation of light depends on the total attenuation coefficient, *µ*_total_ (1/cm), which is the sum of the absorption coefficient of the absorbing species, *µ*_a_, and the reduced scattering coefficient of the scattering species, $\mu_{s}^{\prime }$ (Equation (2)). The total attenuation coefficient represents how strongly light is attenuated by molecular species for a specific wavelength. The reduced scattering coefficient, $\mu_{s}^{\prime }$ is a contribution of the scattering coefficient, *µ*s and the anisotropy of light propagation in biological tissue, g, which is the average cosine of the scattering angle [73]:

(2) $$\mu_{total}=\mu_{a}+\mu_{s}^{\prime }=\mu_{a}+\mu_{s}\left(1-g\right),$$

If *g* is close to 1, more light is scattered in the forward direction compared to backward scattering. The value of g for biological tissue is found to be between 0.65 and 0.95 [73]. This indicates that when light interacts with tissue components, light tends to scatter in the forward direction for a single scattering event. However, after multiple scattering events, the overall light scattering can result in backward scattering. Light scattering due to tissue interference causes glucose measurement errors since light scattering by different individuals varies as a result of the range of tissue fat found in each. In addition, variations in light scattering can be due to differences in collagen, protein, blood flow and hydration state of a person [18,26]. 

It is important to reduce light scattering, and therefore, it is possible to select the wavelength of the light source to have less scattering. The intensity of scattered light due to tissue components is negatively correlated with the wavelength of incident light as described in [50] and is reduced by increasing the wavelength of incident light [50]. As a result, NIR light results in more scattering by tissue compared to MIR light [51]. However, NIR results in higher penetration depth through tissue, compared to MIR.

Diffusion approximation and Monte Carlo modeling are commonly used to simulate light propagation in scattering media such as tissue [54,74]. Scattering particles in tissue cause light to get scattered multiple times and become diffuse. The intensity of diffuse light reflected from tissue depends on the optical properties of tissue which themselves depend on glucose concentration. Equation (3) describes the relationship between optical properties and the intensity of diffuse reflection light in an infinite scattering medium [74]:

(3) $$I\left(\rho \right)=I_{0}\frac{1}{4\pi \rho D}.e^{\left(-\mu_{eff}.\rho \right)},$$

where I(ρ) is diffuse reflection light intensity, $I_{0}$ is incident light intensity, ρ is the radial distance between the light source and a detector (or light source detector separation), and D is the diffusion coefficient which is equal to ${\left[3\left(\mu_{a}+\mu_{s}^{\prime }\right)\right]}^{-1}$ [74]. Based on Equation (3), diffuse reflection light intensity is proportional to the negative exponent of effective attenuation coefficient ($\mu_{eff}={\left[3\mu_{a}\left(\mu_{a}+\mu_{s}^{\prime }\right)\right]}^{1/2}$). Equation (3) was further refined in [75] to process diffused spectral data and improve the accuracy of glucose measurement.

The presence of scattering components in a sample causes multivariate non-linear relationship between absorption spectra and concentration of absorbing components [76]. Various calibration methods such as local regression, artificial neural networks (ANN) and support vector machine (SVM) have been developed to model this non-linearity and improve the accuracy of blood glucose reading. The non-linearity can be minimized by (1) identifying the optimum path length followed by light beam; (2) combination of multiple acquisitions and (3) applying preprocessing algorithms on the spectral information such as multiplicative scatter correction (MSC), orthogonal signal correction (OSC) and net analyte preprocessing (NAP) [76,77].

A particular technique to measure the absorption of light by glucose molecules without being concerned about the interference effect of scattering components is photoacoustic spectroscopy. In this method, a modulated NIR/MIR light beam is irradiated onto a tissue sample where the light is absorbed by absorbing components (such as glucose) and scattered by tissue scattering components. Absorption of light by the absorbing molecules produces a thermal wave that propagates through the sample toward the sample surface. The thermal wave from the sample surface expands to the adjacent boundary gas (gas within the photoacoustic cell), which results in an acoustic signal that can be detected by an acoustic detector. The intensity of the acoustic signal depends on the sample’s absorption coefficient which itself depends on the concentration of absorbing molecules (i.e., glucose). The portion of the light scattered by the scattering molecules has no contribution to the acoustic signal being generated. Thus, photoacoustic based glucose measurements are not influenced by the interference effect of the scattering components. Glucose sensors based on this technique have demonstrated promising results, but are not yet commercially available [78,79,80].

#### 3.1.4. Temperature Fluctuation in Tissue

Another factor affecting the accuracy of glucose reading is temperature fluctuation which affects optical absorption measurements [16,81]. Temperature can be measured using a temperature sensor such as a tympanic membrane thermometer. This sensor is commonly used to determine the temperature of human body based on the intensity of electromagnetic radiation emitted by body at a wide spectral range between 8 to 14 µm [71]. The tympanic membrane is a suitable site for measuring body temperature. Tympanic shares its blood supply with the hypothalamus where is the center for regulation of body‘s core temperature. Measured body temperature should be included in the algorithms, which will help to predict an accurate blood glucose level [81]. Hayter et al. proposed a model to compensate for the effect of skin temperature on glucose measurement as shown in Equation (4) [82]:

(4) $$I_{TC}=I1.07^{\left(32.5-T\right)},$$

where *I* is the raw sensor current signal, *T* is measured skin surface temperature in degrees Celsius and *I_TC_* is temperature compensated current signal that is proportional to glucose concentration. Human skin temperature is normally ranged between 30 °C and 35 °C. However, change in ambient temperature may influence the temperature that is monitored using the temperature sensor on the skin [82]. This effect can be compensated using a second temperature sensor that is placed on the sensor circuit board in order to monitor environment temperature, and including environment temperature in the glucose prediction model [82].

### 3.2. Polarimetry

**Figure 6:**
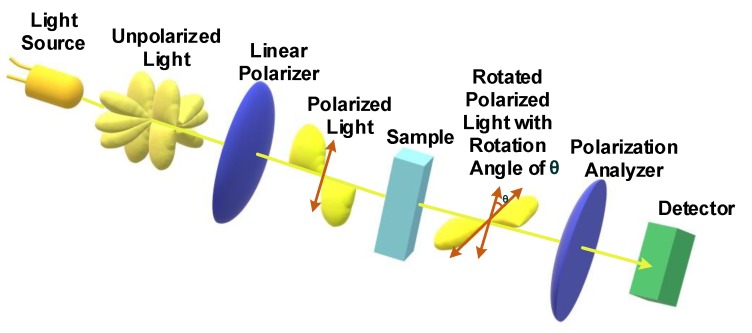
A simplified schematic illustrating polarimeter.

Polarimetry uses linearly polarized light and measures the angle of rotation of the electric field as the light passes through an optically active solution, including glucose in solution. Figure 6 includes a schematic of a polarimeter and its components, such as light source, a linear polarizer, sample, polarization analyzer and photodetector. Un-polarized light is characterized as an electric field that oscillates in many planes with respect to its propagation axis. An ideal linear polarizer can filter the light source so that the electric field within the light source oscillates along only one plane, in this case, perpendicular to the surface of the sample. As it does so, it completely blocks the remaining light intensity in other planes. The active solution affects the angle of the electric field as it passes through the solution. Thus, polarized light passing through a sample containing glucose molecules will result in a rotation of the angle of the electric field from its original angle. A polarizer is also used as a polarization analyzer to determine the plane of the polarized light after it passes through the sample. When the axis of polarization in the analyzer matches the angle of rotation (θ) of the electric field, then the maximum intensity of light will be detected by the photodetector. On the other hand, the photodetector will not detect light when the polarization axis of the analyzer is perpendicular to the angle of rotation of the electric field.

The angle of rotation of the electric field is dependent on the concentration of glucose in the optically active aqueous solution and is modeled by Equation (5) [83]:

(5) $$\theta =\alpha_{\lambda }^{T}l\;c,$$

where θ is the angle of rotation ° of the electric field, $\alpha_{\lambda }^{T}$ is the specific rotation for the active substance (° mL)/(dm g), l is the optical path length within active aqueous solution (dm), and c is the concentration of the active substance (g/mL). The value of the specific rotation of an active molecule, $\alpha_{\lambda }^{T}$, depends on the wavelength of the light source and the temperature of the sample. The angle of rotation of the electric field is shifted in the clockwise direction when light passes through the sample of glucose solution.

Drude’s equation is a known equation used for calculation of specific rotation. Based on Drude’s expression, the value of $\alpha_{\lambda }^{T}$ for glucose molecule is reduced from approximately 74 to 30 (° /mL)/(dm g) when the wavelength of incident light increases from 485 nm to 735 nm at a temperature of 20 °C [84].

One of the difficulties associated with glucose sensing in biological tissue is multiple light scattering, which results in changes in polarization vector orientation and depolarization of light. Thus, glucose concentration within the tissue cannot be measured accurately. 

A possible solution to measure glucose in scattering media is developing a Mueller matrix polarimetry system to extract optical rotation angle and depolarization properties of a sample [85,86]. Mukherjee et al. developed Mueller matrix polarimeter with measurement precision of 0.004° and 0.0004 in optical rotation and depolarization, respectively, and achieved glucose detection sensitivity as low as 20 mg/dL [85]. Another approach to reducing glucose prediction error caused by scattering in slightly turbid media (with scattering coefficient of 0.225–0.275 cm^−1^) is using the Faraday-modulated polarimeter set up and measuring the ratio of light intensity at two specific frequencies [87].

Another promising solution is applying the polarimetric technique on the anterior chamber of the human eye to measure glucose within the humor aqueous. The anterior chamber of the eye is a preferable anatomic location compared to using skin tissue since there are relatively minimal light absorption, minimal scattering and direct correlation between blood glucose and that within the humor aqueous. Glucose within the aqueous humor is about 70% of that in the blood and causes optical rotations in the millidegree range [88]. Polarimetric based glucose measurement in the humor aqueous of eye faces some issues described below along with possible solutions to the issues.

#### 3.2.1. Variation in Corneal Birefringence of the Eye

The birefringence of cornea affects polarization and optical rotation of the measuring beam. The birefringence of the cornea is often varied in time by the presence of movements in the eye [89]. There are permanent movements (micro-tremor and micro-saccades) in the human eye caused by eye muscles and slow movements due to breathing [90]. The frequency range of these movements is between 0.5 and 100 Hz [90].

Time-varying corneal birefringence confounds the polarization state of the beam and interferes with polarimetric based glucose measurement. Furthermore, corneal birefringence varies significantly among individuals [91]. The variation of corneal birefringence between individuals produces differences in the optical polarization state of the beam. So, it is necessary to differentiate between the optical activity of glucose and the birefringence of the cornea. Equation (5) describes the angle of rotation as a function of glucose concentration in a clear optical media. However, the effect of corneal birefringence is not included in this equation. Different models have been developed to facilitate the understanding of the corneal birefringence effect on polarization states of measuring beam [92]. Among them are stromal lamella stacks based model [93], uniaxial model, biaxial model, Navarro eye model [88], and etc. Several methods have been used to extract information about the polarization properties of a sample like aqueous humor, and are such as 16-element Mueller matrix ellipsometry [90,94] and Jones matrix [88,95].

Corneal thickness increases gradually from the center of the cornea (with a mean value of about 0.53 mm) toward the periphery of the cornea (with a mean value of about 0.7 mm) [90,94]. Corneal birefringence depends on the location where the light passes through the cornea and also the angle of incident light [90,92]. The effect of corneal birefringence on the angle of the polarization axis is minimized when beam light passes in the region of 1.5 to 1.8 mm from the apex of the cornea [92]. Another possible solution to minimize the effect of corneal birefringence is improving the speed of polarimetric measurements around 10 ms or less. Yu et al. achieved faster speed by developing a dual modulation-dual wavelength polarimetric system using both laser intensity modulation and Faraday polarization modulation for each wavelength [95]. Error in glucose prediction was reduced from 17.9 mg/dL to 13.9 mg/dL owing to the increase in the speed of the dual-wavelength polarimetric system [95,96].

#### 3.2.2. Presence of Active Components within the Aqueous Humor

Glucose, albumin and ascorbic acid are three main optically active components in the aqueous humor. Albumin and ascorbic acid can affect the angle of rotation independent of the glucose concentration [89]. Baba et al. demonstrated that adding albumin and ascorbic acid to a glucose solution results in a decrease and increase in the angle of rotation, respectively [97]. So, these two components partially cancel the effect of each other on the angle of rotation. However, their concentration variation between individuals may cause errors in glucose prediction. Contribution of albumin and ascorbic acid can be minimized when using a light source with a higher wavelength between 600 and 750 nm in comparison to a lower wavelength between 300 and 600 nm [97].

Using multiple wavelengths can help to minimize the measurement error due to albumin and ascorbic acid. Each molecule results in a specific angle of rotation for each wavelength and as a result, the optical rotatory dispersion curve is unique for each molecule.

#### 3.2.3. Lag Time between Blood Glucose Measurements in Blood Plasma vs. Aqueous Humor

A disadvantage of using the polarimetric method for glucose monitoring of humor aqueous is the physiological lag time (below ten mins) between the peak of glucose in humor aqueous and the associated glucose peak measured in blood plasma, that prevents from a real-time measurement. Purvinis et al. obtained the lag time in the range between 2.9 and 5.4 min based on measurements of glucose concentration within the anterior chamber of the eye for New Zealand white rabbits [98]. In this experiment, a Faraday-based polarimeter was used at a red light wavelength to measure the angle of rotation with a sensitivity below 0.4 millidegrees, which corresponds to less than 10 mg/dl of glucose concentration. In the case of human eye glucose measurements, a lag time of 4 min to 7 min was estimated using a mathematical model described in [90]. However, the lag time associated with glucose measurements needs to be more investigated.

#### 3.2.4. Temperature Fluctuation

Results obtained via polarimetry are affected by temperature of the solvents. The specific rotation for glucose molecules increases with temperature for a given wavelength and pH [97]. The contribution of temperature to the rotation of polarized light decreases when the wavelength of incident light increases from 350 to 750 nm [97].

### 3.3. Raman Spectroscopy

**Figure 7:**
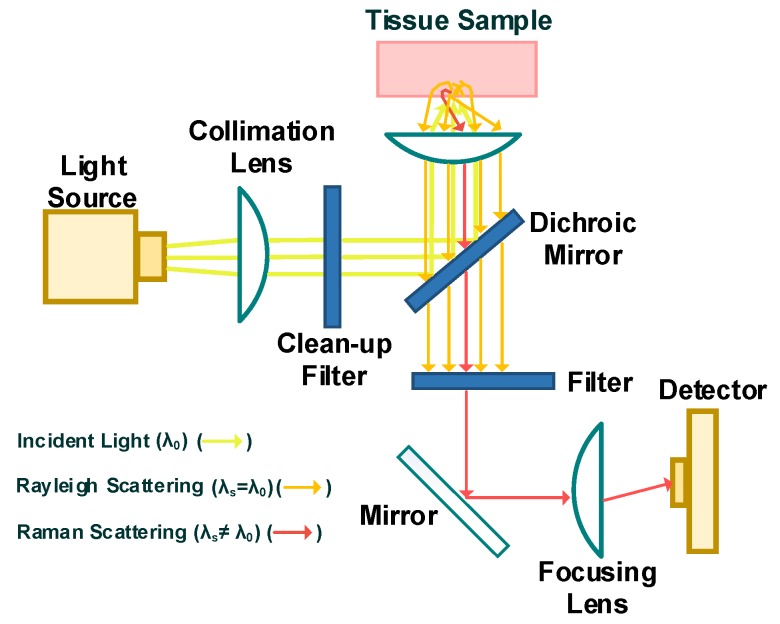
A simplified schematic illustrating Raman spectroscopy.

The Raman spectroscopy setup includes a high intensity light source, mostly in the NIR range, and a very sensitive Raman spectrum photodetector. Figure 7 is a simple schematic representation of the Raman spectroscopy. When light with a monochromatic frequency interacts with matter, a portion of the light is scattered. The scattering is mostly elastic (such as Rayleigh scattering and Mie scattering), with a very small percentage of inelastic including Raman scattering (stokes and anti-stokes). Elastic scattering produces light with the same frequency and wavelength as the incident light, while inelastic scattering generates light with multiple wavelengths and frequencies [99]. Most of the light that is scattered is elastic, and about one in every million scattered photons takes part in inelastic scattering [48,100]. The Raman schematic in Figure 7 illustrates how the dichroic mirror and filter selectively suppresses the light scattered from the sample and eliminates a portion of the elastically scattered light (here Rayleigh light) prior to reaching the detector. 

In Raman scattering, the change in wavelength and frequency between the incident and emitted light is due to the interaction of light with the scattering molecules. Electrons within the molecules absorb enough energy from the incident light to transition to higher energy states, and when the electrons transition back down to different energy levels, the transitions result in the emission of photons with frequencies that differ from those of the incident photons, thus characterized as Raman shift. Raman shifts are expressed as wavenumbers with units of cm^−1^. The energy transitions are associated with the vibrational and rotational energy levels, and in the case of infrared incident light, these transitions are associated with vibrational energy levels. The molecules of a given material have specific vibrational energy levels, and therefore, produce a unique Raman fingerprint, which includes a spectrum of scattered light (Raman) along with incident light (Rayleigh). The peak height, or intensity of the Raman spectrum, is dependent on the substance concentration and the wavelength of the light source, with an increase in the intensity of the Raman peaks as the incident wavelength is decreased [6,28]. The shift in the frequency of the scattered light depends on the type of molecules or the chemical structure of the sample, and is independent of the wavelength of the light source.

The vibrational modes for glucose molecules (C_6_H_12_O_6_) are associated with C-O, C-C and C-H stretching bonds and are observed between 800 and 1200 cm^−1^ for C-O and C-C and around 2900 cm^−1^ for C-H [101]. Thus, typical glucose Raman fingerprints are observed at 911, 1060 and 1125 cm^−1^, with the highest intensity Raman signal at 1125 cm^−1^ [28,102].

Non-invasive glucose Raman-based detection is possible with calibration stability of at least 10 days [102]. The measurement set up for this system includes an 830 nm light source irradiating at 250 µm below the skin surface (targeting the interstitial fluid region) of the thumb in 35 patients. It was demonstrated that 93% of measured data points were placed in the region of A + B using consensus error grid analysis.

There are obstacles to accuracy and precision associated with Raman-based non-invasive glucose measurements. Several physiological factors that affect Raman-based measurements of glucose are explained below.

#### 3.3.1. Water and Other Blood Constituents

The Raman spectrum of water has a weak cross-section, and therefore, has a minimal effect on the glucose Raman spectroscopy measurement. The large Raman shift due to the OH stretching bond is 3400 cm^−1^ and can be measured to estimate the water content of blood [103]. The blood Raman spectrum includes obvious peaks at 650, 758, 837, 945, 978, 1004, 1130, 1163, 1217, 1332, 1551 and 1660 cm^−1^ [104]. Glucose makes up a small percentage of the total blood volume and thus makes up a small portion of Raman peak amplitudes. The analysis of more than one substance in blood results in multiple peaks and multivariate analysis of the spectra is required to extract glucose information from the Raman spectra. Analysis methods include partial least squares regression (PLS), principal component analysis (PCA), support vector machine (SVM) and backpropagation artificial neural network (BP-ANN). The first two methods can be applied as data compression techniques and the two latter methods can be used for finding both linear and non-linear relationships between the glucose concentration and the measured spectrum [102,104,105,106].

#### 3.3.2. Tissue Variation between Individuals

Skin tissue characteristics vary between individuals and tissue characteristics may affect the measured intensity of the Raman fingerprint of glucose. Various methods exist to overcome errors and/or barriers associated with the measurement of blood glucose surrounded by tissue and body fluids within the body.

- Signal filtering applied by multivariate analysis of Raman spectra from multiple blood/tissue components (as mentioned in Section 3.3.1) and data calibration applied to a glucose prediction model using a fraction of the total data followed by validation of the rest of the data as independent test data [102,104,105,106].
- Normalization of glucose Raman intensity peak with respect to a more stable reference within the body, such as hemoglobin. Hemoglobin concentration does not vary significantly between individuals [28]. Consequently, the relative Raman intensity of glucose is the glucose Raman measurement normalized to the Raman fingerprint of hemoglobin at 1549 cm^−1^.
- Selection of a test site with a nearly transparent epidermis and a high density of blood vessels. The nail fold or volar side of the fingertip are good examples that minimize signals from tissue components and maximize Raman spectra from blood components [104,107]. Selecting a measurement site with a high density of blood vessels minimizes the time lag between actual blood glucose measurements vs. glucose within the tissue.
- Tissue modulation optimizes the signal originating from blood components vs. tissue components [106].
- Use of an actuator to apply controllable pressure to the measurement site in order to improve reproducibility [107].

#### 3.3.3. Fluorescence Signal due to Presence of Protein

The presence of proteins in blood also has an effect on the Raman spectra that is dependent on the light source. Proteins emit a background fluorescence signal due to a series of electron transitions between two singlet states [108]. The intensity of the background fluorescence signal is equal to or larger than the Raman signal [16]. A reduction in the protein fluorescence signal is possible by using a longer wavelength (red or lower end of near-infrared) as the light source [100,109].

A small change in the light source wavelength will not affect the fluorescence spectra significantly, while the Raman fingerprint will shift [103]. Thus, two light sources with two similar wavelengths are used and the removal of one signal from the other will eliminate the unwanted fluorescence background signal present in both signals, while leaving the Raman glucose spectra.

An alternative solution to avoid protein interference in skin tissue is to measure the glucose concentration within the anterior chamber of the eye. Eye measurements require a lower power irradiation light source, however, this results in a lower signal to noise ratio.

#### 3.3.4. Inherently Weak Raman Signals

The signals associated with Raman scattering are weak in comparison to elastic scattering intensity peaks. The analysis of blood glucose by the Raman spectroscopy method is also a challenge due to the low percentage of glucose among other biological components in blood. There are several methods to enhance the sensitivity of Raman spectroscopy, such as surface enhanced Raman spectroscopy (SERS). SERS, developed in the mid-1970s, is due to a localized surface plasmon generated by an electromagnetic resonant effect between a substrate and an excitation light source across the low concentration analyte. This effect generates an increase in signal by 11 orders of magnitude [100,110]. Metal (ex. gold, silver and copper), semiconductor and quantum dot substrates are designed and fabricated with design parameters that maximize the localized surface plasmon resonance effect [109]. The substrates can also be treated and/or coated with linker molecules to maximize the glucose affinity to the substrate [111,112]. Glucose measurements using SERS within the interstitial space between the muscle and dermis of rats and through a contact lens mounted on an artificial glass eye have been successful [109,113]. SERS measurements as a function of Raman peak shift (versus Raman peak intensity) have shown similar results [111]. Although SERS based glucose sensors show promise, more work is required before applying these techniques to in vivo glucose measurements in humans.

## 4. Non-Invasive Glucose Sensing Methods Based on Tissue Properties

Optical and electrical properties of tissue and blood are a basis for some of the non-invasive glucose measurement methods. The tissue scattering coefficient and blood refractive index are two optical properties whose values depend on glucose concentration. Two non-invasive glucose detection methods, scattering/occlusion spectroscopy and optical coherence tomography, function based on these two values. The tissue permittivity and conductivity are electrical properties of tissue which are also sensitive to glucose concentration. This dependency creates a basis for two other non-invasive methods. Bioimpedance spectroscopy and millimeter-wave/microwave/ultra-high frequency wave sensing (mmW/MW/UHF sensing) function based on both these properties.

This section focuses on non-invasive glucose measurement methods based on optical and electrical tissue characteristics.

### 4.1. Scattering and Occlusion Spectroscopy

**Figure 8:**
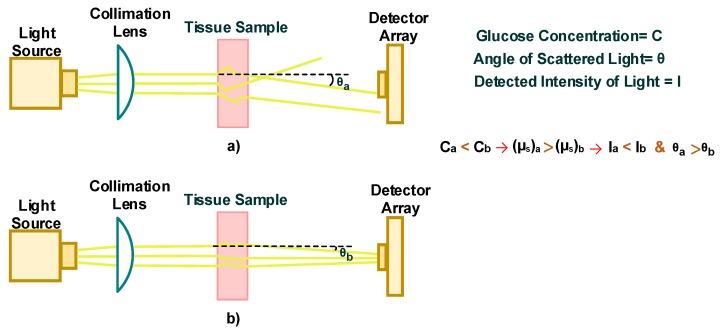
A simplified schematic illustrating scattering spectroscopy of a (**a**) low glucose concentration tissue sample versus a (**b**) high glucose concentration tissue sample.

Scattering spectroscopy measures glucose concentration based on the scattering property of light within the tissue. Figure 8 includes a schematic of a scattering spectroscopy set-up with a red or near-infrared light source and a photodetector array illustrating the scattering behavior of a tissue sample at (a) low glucose concentration and (b) high glucose concentration. In the case of the low glucose concentration, Figure 8 demonstrates that the scattering angle exceeds that of the high glucose concentration sample, and thus the intensity of the scattered light at the detector for the low concentration sample is less than that of the high glucose concentration sample.

There are several parameters used to model the scattering of light by glucose molecules in the body. One of these is the reduced scattering coefficient of tissue ($\mu_{s}^{\prime }$), which is affected by glucose concentration. The reduced scattering coefficient of tissue depends on the mismatch between the refractive index of the extracellular fluid and the refractive index of tissue scatterers (membranes of the tissue cells and cellular components) [114]. An increase in plasma glucose concentration, increases the refractive index of the extracellular fluid (n_m_), while it is assumed that the refractive index of the cellular membrane (n_s_) remains relatively constant [115]. It is reported that the value of n_m_ increases by 1.52 × 10^−5^ for each 10 mg/dl increase in glucose concentration [116,117]. Change in n_m_ causes changes in the scattering properties of particles suspended in the blood such as red blood cells which are occupying around 45% of the blood volume.

Equation (6) is a simple model that describes the behavior of the reduced scattering coefficient of tissue as a function of *n_m_* and *n_s_* [33]:

(6) $$\mu_{s}^{\prime }=3.28\pi r^{2}\rho_{s}{\left(\frac{2\pi r}{\lambda }\right)}^{0.37}{\left(\frac{n_{s\;-\;}n_{m}}{n_{m}}\right)}^{2.09},$$

where *r*, $\rho_{s}$, *λ* are the radius of the scattering sphere, the volume density of the spheres and the wavelength of the incident light, respectively. Based on Equation (6), an increase in the refractive index of the extracellular fluid (*n_m_*), results in a decrease in the refractive index mismatch (*n_s_*–*n_m_*), which results in a reduction of the reduced scattering coefficient of the tissue $\mu_{s}^{\prime }$. Equation (6) is valid for non-interacting Mie scatterers (tissue particles in which the size of the particles involved in scattering is comparable to the wavelength of the incident light), and assumes that the anisotropy of light propagation in biological tissue (g) is greater than 0.9, that 2*πr*/*λ* is between 5 and 50, and that the ratio of n_s_/n_m_ is between 1 and 1.1 [33]. In the NIR range, the values of *n_m_* and *n_s_* are between 1.348–1.352 and 1.350–1.460, respectively [118,119].

Beer’s law ($I=I_{0}e^{\left(-l.\mu_{a}\right)}$), states that light attenuation due to scattering is proportional to the negative exponent of the absorption coefficient ($\mu_{a}$) in a case that $\mu_{s}^{\prime }$ is negligible. The values of $\mu_{a}$ and $\mu_{s}^{\prime }$ of the sample also depend on the wavelength of the incident light. Light scattering in tissue is dominant at lower wavelengths, i.e., in the NIR window, compared to the MIR range [120]. To more accurately describe light intensity correlation with scattering properties of tissue, other common theories are required such as Rayleigh theory, Mie scattering theory, diffusion theory and Monte Carlo simulation [33,121,122].

Occlusion spectroscopy is a form of scattering spectroscopy that relies on the deliberate application of pressure at the tissue site. Pressures above the systolic pressure obstruct blood flow that causes an increase in the agglomeration of red blood cells and create an effective increase in the average size of scattering particles. This results in a dynamic change in blood flow and an increase in the intensity of the detected scattered signal along with enhanced sensitivity to changes in the glucose concentration [18,123].

The OrSense’s NBM-200G device (OrSense Ltd., Nes Ziona, Israel) is an example of a commercial non-invasive glucose sensor based on occlusion spectroscopy. The testing of the sensor on the fingertips of 12 T1D and 11 T2D patients resulted in 95.5% of the measurement data within the clinically acceptable A (69.7%) and B (25.7%) regions of the Clarke error grid analysis chart [123]. Although the OrSense has a CE safety designation, it is not commercially available at this time.

Abdalsalam and Awouda designed an occlusion spectroscopy system using a NIR source and a linear position detector to measure light intensity as well as the angle of scattered light transmitted through the index finger [122]. The Rayleigh theory was used to calculate glucose concentration based on the measured light intensity and the angle of scattered light. The clinical accuracy of the glucose measurements for 55 volunteers was 72.7% and 27.3% in regions A and B, respectively, on the Clark error grid analysis chart [122].

Sun and Chen proposed a combination of occlusion spectroscopy and time-resolved spectroscopy to enhance the sensitivity of glucose measurements [121]. In this study, a single layer finger model consisting of blood plasma and red blood cells is used. Two wavelengths of 610 nm and 810 nm are the light sources and a time-resolved optical detector measures light transmitted through the sample. The ratio between the incremental changes in the optical transmittances at the two wavelengths is defined as the parametric slope. Modified parametric slopes are derived from Laplace transformed time-domain data. A strong correlation was observed between the modified parametric slopes and glucose concentration [121]. However, the suitability of this approach for in vivo measurements needs to be investigated.

There are physiological factors that affect the accuracy of glucose measurements based on the occlusion and scattering spectroscopy methods. Variations in the free fatty acid concentrations, oxygen saturation and intrinsic erythrocyte aggregation within the tissue all affect the scattering of light [18,124]. The effect of these interfering components can be minimized by using light sources with multiple wavelengths and by applying a suitable and sophisticated algorithm to extract glucose information from multispectral data [123,125]. The next three subsections explain other factors that affect the accuracy of glucose measurements.

#### 4.1.1. Blood Protein Variation between Individuals

Zirk and Poetzschke investigated blood glucose concentration measurements based on the blood refractive index using a commercial refractometer [26]. The effect of protein on the refractive index is significant since protein is the second dominant species in blood plasma, which equates to a dominant contribution to the blood refractive index compared to that of glucose. Zirk and Poetzschke reported a positive correlation of 0.973 between the refractive index of ultra-filtrated blood and the respective glucose levels of four non-diabetes patients [26]. Filtration includes the removal of high molecular size substances, such as lipoproteins and proteins. Without filtration, there is no clear correlation between the refractive index and the blood glucose concentration. This is due to a significant effect of protein on the total blood refractive index. The presence of protein interferes with the glucose measurement, and this is complicated by the variation of protein levels between individuals.

#### 4.1.2. Blood Osmolality Variation between Individuals

The normal range for blood serum osmolality, dissolved non-electrolyte molecules and ions, is between 285 and 293 mmol/L. In [26], the effect of blood osmolality on the accuracy of glucose measurements was investigated in a non-diabetic human subject as a function of the blood refractive index. The subject was tested under two conditions, the restriction of water intake which increases serum osmolality, and the ingestion of water which decreases blood serum osmolality. The glucose concentration measurements demonstrated a relative deviation of more than 50% between the measured values of glucose concentration using the Accutrend Sensor (Roche Diagnostics, Manheim, Germany), versus the refractometry method for osmolality values of 273 mmol/L and 296 mmol/L.

Friebel et al. measured optical parameters of red blood cells in a saline solution and demonstrated that variation in osmolarity causes a significant change in the value of $\mu_{s}^{\prime }$ [124]. A relative $\mu_{s}^{\prime }$ with the value of 0.65 ± 0.06 was measured for an osmolality of 225 mmol/L while 1.39 ± 0.06 was measured for an osmolality of 400 mmol/L over a spectral range between 600 and 1100 nm.

#### 4.1.3. Variation in Skin Scattering Coefficient due to Age and Sex

A non-invasive Periflux 6000 Enhanced Perfusion and Oxygen Saturation (EPOS) system (EPOS, Stockholm, Sweden) as used to measure the reduced scattering coefficient ($\mu_{s}^{\prime }$) on the volar forearm of 1734 human subjects (men and women between the ages of 50 and 64 years) and data was analyzed with an inverse Monte Carlo algorithm [50]. Based on this study, the mean value of $\mu_{s}^{\prime }$ decreases from 3.16 mm^−1^ at 475 nm to 1.13 mm^−1^ at 850 nm. The results of this study also indicate that $\mu_{s}^{\prime }$ has a lower value in women compared to men and that there is a reduction in $\mu_{s}^{\prime }$ with age. A reduction in the collagen level during aging and a lower level of collagen in women may be responsible for these observations since collagen acts as a main scattering mechanism in the dermis layer [50,126,127]. Therefore, collagen variation can interfere with the accuracy of glucose sensing due to its influence on the value of $\mu_{s}^{\prime }$.

### 4.2. Optical Coherence Tomography

**Figure 9:**
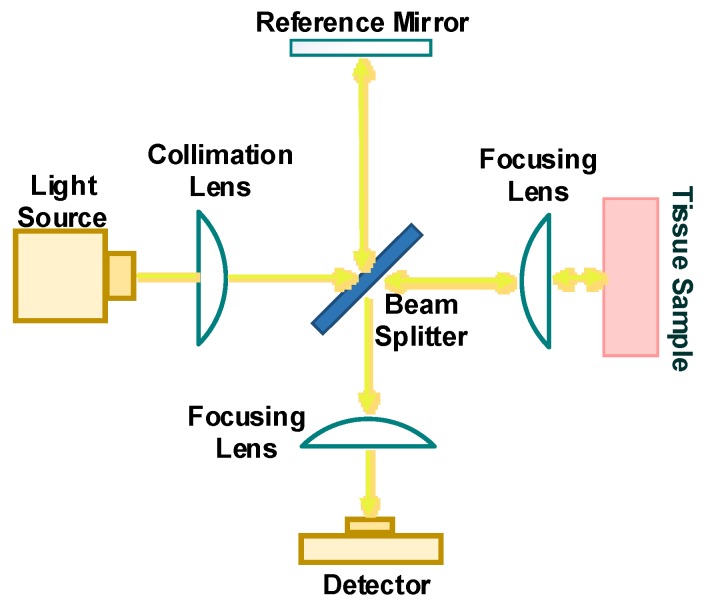
A simplified schematic illustrating optical coherence tomography of tissue.

Optical coherence tomography (OCT) is a high-resolution optical imaging technique. The OCT system uses a low coherence light source within the NIR range, with a coherence length between 10 and 15 μm, with an interferometric signal coming from the tissue sample and a reference mirror. Figure 9 includes a schematic of an OCT system, which includes interferometer optics and a photodetector/camera set-up. A light source is split into two beams, one is backscattered from the tissue sample and the second beam is reflected from a reference mirror to the beam splitter [51,73,114]. The combination of light returning from the sample and the reference mirror results in the interferometric signal at the beam splitter. The photodetector collects the interference pattern and the measured intensity is dependent on the glucose concentration at different tissue depths, up to 1.6 mm [128].

The OCT signal can be measured at a specific depth of the tissue layer by scanning the mirror in the reference arm, without any interference from other tissue layers. Using a second moving mirror into the tissue sample arm allows scanning of probing beam laterally over the tissue surface, so there will be two-dimensional images in both the lateral and in-depth [33,114,129]. The OCT technique has the capability to obtain microstructure imaging with high signal to noise ratio up to 130 dB [130]. So, OCT is capable to detect very low light intensity scattered back from biological tissue. The tissue scattering coefficient (µ_s_) is dependent on the glucose concentration, and as the glucose concentration increases, the refractive index of the extracellular fluid increases and thus the tissue scattering coefficient decreases. Therefore, as the glucose concentration changes, so does the intensity of the backscattered light reflected from the tissue sample layers.

The relationship between the intensity of the OCT signal and the glucose concentration can be modeled by Equation (7), where the square of the intensity is related to the tissue scattering coefficient [73,131]:

(7) $$I^{2}\left(l\right)=rI_{0}^{2}\frac{\mu_{b}\left(l\right)}{4\pi }Le^{-2ln\mu_{s}},$$

where *r* is the reference reflection coefficient, *I*_0_ is the intensity of the incident light, *L* is the temporal coherence length of the incident light, *n* is the mean refractive index of the tissue (≈1.38), and *l* is the penetration depth. The parameter *µ_s_* is the wavelength-dependent scattering coefficient, and the parameter *µ_b_* is the backscattering coefficient after the light has penetrated the tissue a depth equal to *l*. Light attenuation due to the scattering of light is dominant at lower wavelengths. Therefore, wavelengths in the NIR range are used in order to maximize scattering. The value of $\mu_{a}$ = 1 cm^−1^ and $\mu_{s}^{\prime }$ = 100 cm^−1^ were widely accepted for tissue simulating phantoms in the range of NIR [132].

The OCT signal intensity is mostly a function of distance from the surface of the skin. The slope of a straight line fitted to the OCT signal depth profile depends on glucose concentration. The correlation coefficient between glucose concentration and the OCT signal slope varies periodically (with a period of 100–150 µm) between −0.9 to 0.9 depending on the depth where the tissue layer is scanned [133]. The OCT measurement on 15 healthy subjects demonstrated that the OCT signal slope decreases up to 2.8% per 10 mg/dL increase in plasma glucose concentration when the slope of the OCT signal is measured at a depth between 200 and 600 µm from the skin surface [114]. In vivo experiments on farm pigs show the maximum correlation between glucose concentration and the OCT signal slope at the papillary-reticular and dermis-hypodermis junctions [134].

Like other methods, the OCT based glucose measurement faces challenges associated with several physiological and experimental conditions.

#### 4.2.1. Tissue Heterogeneity & Scattering of Light by Tissue

The specificity of the OCT method for non-invasive detection of blood glucose is affected by the scattering of light due to water, red blood cells (RBCs), fat, collagen fibers and proteins (such as keratins in the epidermis) [135]. The presence of these scattering components varies from subject to subject and limits the light penetration depth. For example, the range of sizes of RBCs in the blood varies between each human subject. The presence of a range of scattering components causes a cyclic variation in the OCT signal vs. glucose concentration. This results in a non-linear correlation between the amplitude of the OCT signal and the glucose concentration for a given depth within the tissue. Furthermore, the average size and size distribution of RBCs are different between each individual. Consequently, it is difficult to predict the glucose concentration with accuracy without a RBC reference size [136,137,138].

In addition, changes in the concentration of osmolytes in the body such as KCl, Urea and NaCl can also affect the scattering coefficient of tissue. Although the intensity of the OCT signal is sensitive to concentrations of these components, which can vary between individuals, the effect of glucose on the signal is still dominant [16,33,136,139]. 

Identifying appropriate measurement site is necessary to minimize the effect of multiple scattering on the OCT based glucose measurements. The depth of the dermis layer in the human arm and forefinger is between 166–276 µm and 441–579 µm, respectively [140]. Since the dermis layer thickness in the arm is smaller, it is less prone to multiple scattering effects caused by other tissue components, and this makes it a preferable site for OCT measurements.

Attempts to improve the accuracy of the OCT imaging system are numerous. For example, a theoretical model developed by Thrane et al. is based on the extended Huygens–Fresnel principle for optimization of the OCT system to yield a maximum heterodyne signal [141]. However, all theoretical models require validation by testing on tissue phantoms with scattering and absorption characteristics that match human tissue. Intralipid based optical phantoms are an optimal choice for validation of the theoretical/experimental system [142].

A second approach is to increase sensitivity to glucose by implanting a glucose recognition unit under the skin [143,144]. A third solution to improve the selective detection of glucose is a combined OCT technique with the Mueller matrix polarimetry technique [145] and a combined OCT technique with dual-wavelength absorption based technique [117,146,147]. Measurements made using various combinations of OCT methods have resulted in detection limits between 2.4 and 69.6 mg/dL for glucose within the anterior chamber of the eye model [117,147].

#### 4.2.2. Patient Motion Artifacts

Motion artifacts during the OCT measurements have an effect on the OCT signal slope and the accuracy of the glucose measurement. The motion artifact induced error can be minimized by using a high speed recording OCT system [148,149]. Post processing or using a motion tracking system are two other possible solutions for minimizing the error associated with motion artifacts [150]. An experiment on 19 anesthetized farm pigs demonstrated a reduction in motion artifact induced error and improved reproducibility by placing an OCT probe on the skin with slight controllable pressure (<1 kPa) on the probe [134].

#### 4.2.3. Lag Time between Blood Glucose Measurements in Plasma vs. Interstitial Fluid

One disadvantage of the OCT system is the time lag between a change in the OCT signal slope and the actual change in blood glucose levels [33]. This physiological time lag is between a few seconds and 15 mins [23]. Lan et al. took OCT measurements from 6 diabetic patients’ forearms using a wavelength of 830 nm [128]. The depth region of 320–460 µm was used to calculate the OCT signal slope. A time lag of 10 mins was observed between a change in blood glucose concentration and an associated change in the OCT signal slope [128]. In another experiment, a time lag of 1–30 min was measured between the OCT signal slope variation and the 225–389 mg/dl change in blood glucose levels of ten female pigs [139]. Measuring the OCT signal at a depth where there is high vascularity in the tissue helps to reduce the time lag associated with this type of measurement.

#### 4.2.4. Temperature Fluctuation in Tissue

Cooling and heating the skin tissue results in a decrease and increase, respectively, of the tissue reduced scattering coefficient. The light penetration depth increases at lower temperatures due to a lower tissue scattering coefficient [120]. Skin thickness also fluctuates with changes in temperature. Forst et al. measured the forearm skin thickness of 13 diabetic patients and 7 healthy subjects vs. an ambient change in environmental temperature from 25 °C to 4 °C [151]. An average skin thickness reduction of −0.09 ± 0.13 mm and −0.06 ± 0.11 mm was observed for diabetic and healthy subjects, respectively. Temperature induced changes in skin thickness and $\mu_{s}^{\prime }$ have an influence on OCT signals and thus affect the accuracy of glucose measurements. In [152], the effect of temperature on accuracy of OCT based glucose measurements was investigated in nine healthy subjects. On average, a glucose prediction error of 0.3 ± 0.097 mmol/L was reported as a result of a 1°C temperature fluctuation.

### 4.3. Bioimpedance Spectroscopy

Bioimpedance spectroscopy employs the measurement of impedance levels within tissue using a small AC signal with a frequency below 1MHz. Biological tissue can be modeled as an electric circuit of resistors and capacitors [153]. Capacitance and resistance originate from the cellular membrane structure and the body water fluid (intra and extracellular fluid), respectively [154]. Cell membranes are semipermeable and separate intracellular spaces from extracellular spaces. A simplest electrical model for tissue is a parallel combination of a conductor and capacitor, however, more realistic tissue electrical models have been proposed [153,155,156]. The conductivity of tissue is related to movements of ions within the biological fluid and permittivity is related to the tissue’s ability to store charge or rotate molecular dipoles in the presence of an electric field [153,157]. Equation (8) describes the impedance of tissue based on the simplest electrical model as a function of tissue conductivity σ and permittivity ε [157]:

(8) $$Z=\frac{1}{G+j\omega C}=\frac{d/A}{\sigma +j\omega \varepsilon },$$

In Equation (8), *G* and *C* represent conductance and capacitance of the equivalent model. Parameters *d* and *A* are the thickness and cross-sectional area of the tissue sample, and ω is the angular frequency of the applied signal to the tissue. The impedance of tissue depends on the frequency of the applied signal. At low frequency, the current signal flows only through the extracellular fluid and contributes to the conductance portion of the tissue impedance. However, at high frequency, the current flows through both the intra and extracellular fluid and penetrates the cellular membrane, thus contributing to both the conductance and capacitance part of the tissue impedance [154].

**Figure 10:**
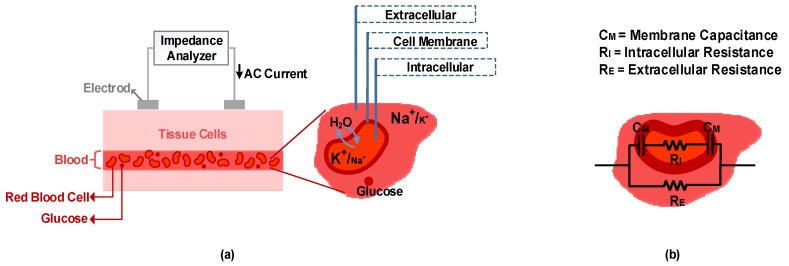
(**a**) A simplified schematic illustrating tissue impedance spectroscopy, (**b**) An electrical model for a single red blood cell.

Figure 10a illustrates the physiological components that play a role in tissue impedance behavior. Tissue impedance depends on the electric characteristics of the cell membranes. Since RBCs suspended in the blood occupy around 45% of the blood volume, the RBC membrane has an important role in the total tissue impedance [158] and has an effect on the capacitance value in the equivalent circuit of the tissue. In a microscopic view, the electrical behavior of a single red blood cell is modeled as illustrated in Figure 10b [159]. The red blood cell membrane contributes to the capacitance, and the intra and extracellular fluid contribute to the resistance of the electrical model. On a macroscopic scale, the electrical model for human tissue is more complex as described in [153,155,156].

When the blood glucose concentration increases, the serum osmolality increases which results in the movement of water out of the cell and into the extracellular spaces through the cell membranes. This causes the sodium ion [Na^+^] levels in extracellular spaces to decreases due to the dilution. Cellular dehydration promotes the redistribution of potassium ions [K^+^] from the intracellular to the extracellular spaces, increasing the serum [K^+^] ion level in extracellular spaces [158,160,161].

As the [K^+^] and [Na^+^] ions are balanced, there are associated changes to the permittivity and conductivity of surrounded medium and cell membranes, including the RBC membrane. These activities result in changes to the tissue impedance [19]. According to a study conducted by Li et al., the permittivity and conductivity of an aqueous solution decrease when the glucose concentration increases for a frequency range between 1 kHz and 1 MHz [162]. Based on Equation 8, the magnitude of the impedance increases with a decrease in the permittivity and conductivity [163,164]. Impedance spectroscopy encounters measurement and accuracy challenges, and these are described below, along with possible solutions.

#### 4.3.1. Tissue Heterogeneity and Variation in Red Blood Cell Morphology

There are differences in tissue components and red blood cell morphology between individuals. This results in different tissue impedance among people regardless of blood glucose level and so, affect glucose measurements while using the bioimpedance spectroscopy method [165]. A possible solution to remove the influence of tissue component interferers is impedance spectroscopy based on blood pulsation during cardiac cycles [162]. Li et al. carried out an in vitro experiment using agar phantom in which the change of blood volume due to heartbeat was simulated [162]. The difference between the minimum and maximum impedance values at each cycle was measured by applying a 25 kHz signal to the phantom. This process was repeated for different glucose concentration and resulted in normalized impedance difference of 1, 0.964, 0.959 and 0.930 Ω for glucose concentration of 0, 50, 100 and 200 mmol/L, respectively. In this research, the influence of blood on measuring impedance is decoupled from the influence of skin/fat tissue components via measuring variations in blood impedance during pulsatile flow.

Another possible solution to improve the accuracy of glucose reading is a combination of different glucose sensing methods. A combination of methods can compensate for the error caused by each method separately and thus minimize the effect of confounding factors. A combination of two methods of (1) scattering spectroscopy at three wavelengths of 850, 950 and 1300 nm, and (2) impedance spectroscopy in the frequency range between 10 and 76 kHz was proposed in [166]. The blood glucose level of ten normal volunteers was estimated based on two methods separately and then the obtained results were combined using an artificial neural network algorithm in order to improve the accuracy of glucose estimation. In this research, the accuracy of 100% was achieved based on the Clarke error grid analysis. The approach of using multiple technologies was employed in a non-invasive glucose sensor device named GlucoTrack which was developed by Integrity Applications Company (Ashdod, Israel). GlucoTrack device is based on a combination of three technology of ultrasound, thermal and impedance spectroscopy. The performance of the GlucoTrack device was evaluated by targeting the ear lobe of 91 diabetes in [167]. It was shown that 96% of the glucose reading fell in the clinically accepted A and B zone using the Clarke error grid analysis.

#### 4.3.2. Patient Motion Artifacts, Sweat/Humidity and Temperature Fluctuation in Tissue

Motion artifacts and humidity/drying at tissue surface act as interferes for tissue dielectric measurement. Another perturbing factor is variation in temperature since electrical characteristic of tissue depends on tissue temperature. Change in impedance level with respect to temperature variations was explored in a study by measuring impedance of a solution consisting of sodium chloride and glucose at 1 MHz [168]. It was shown that increase in temperature from 32 °C to 42 °C make a reduction of about 22 Ω in impedance value of the sample. In order to include the influence of perturbing factor on glucose reading, functional relation between perturbing factors and measured signal (here impedance of sample) can be derived by applying multiple regression on measured data [169].

Multiple sensor technologies provide an opportunity to measure temperature, humidity and motion artifacts by using temperature sensor(s), sweat/moisture sensor(s) and three axes accelerometer, respectively. Geng et al. developed multiple sensors system to measure tissue impedance in six healthy and three diabetes human subjects at frequency range between 1–50 kHz and 10–60 MHz [170]. Using Clarke error grid analysis, 92.86% of estimated glucose level fell in zone A and 7.14% in zone B which means successfully glucose reading.

### 4.4. Millimeter Wave/Microwave/Ultra-High Frequency wave sensing

**Figure 11:**
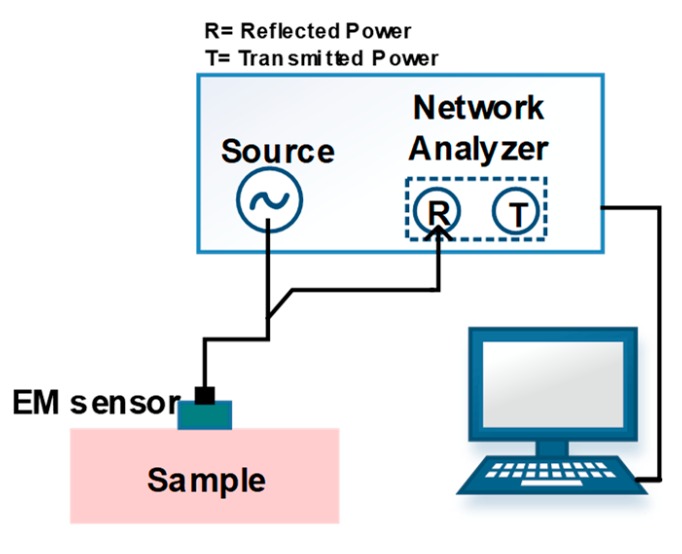
A simplified schematic illustrating a reflection mode EM measurement.

Millimeter waves, microwaves and ultra-high frequency waves are electromagnetic (EM) radiation with a frequency range between 30–300 GHz, 3–30 GHz and 300 MHz–3 GHz, respectively [171]. When EM radiation is applied to tissue, some portion of the EM radiation energy is reflected back from the tissue surface. The rest of EM radiation energy is transmitted through the surface toward underlying layers in which some portion of transmitted signal is absorbed. Absorption, reflection and transmission of EM waves through the tissue depends on the dielectric characteristics, which depend on the glucose concentration. Reflected or transmitted EM radiation is sensed using a single-port or two-port EM wave sensor, respectively. The measured signal is analyzed using a vector network analyzer (VNA) and computer software that extracts glucose concentration information. A simplified schematic for a reflection mode EM measurement device is shown in Figure 11.

The permittivity and conductivity of the tissue depend on the frequency of the applied EM signal and the glucose concentration [1,172,173]. Yilmaz et al. investigated the relationship between the dielectric properties of tissue-mimicking phantom and the frequency of the applied EM signal at a frequency range between 0.3–20 GHz by [172]. The relative permittivity measurement shows a decreasing trend (approximately from 68 units to 22 units), and the conductivity measurement demonstrates an increasing trend (approximately from 2 to 28 S/m) as a result of an increase in the frequency. An overall increasing trend in the conductivity and a decreasing trend in the permittivity was also observed by Gabriel et al. over an increase in frequency between 10 Hz and 20 GHz for different types of human and animal tissue (liver, muscle, skin, tongue, adipose tissue) measured at body temperature [174]. 

The dependency of glucose concentration on relative permittivity was investigated by Jang et al. [1]. In this research, a negative relation between relative permittivity and glucose concentration in a solution was demonstrated over a frequency range between 1 and 15 GHz. At a single frequency of 2.9 GHz, an increase in glucose concentration from 0 to 400 mg/dl results in a total reduction of 0.2 units in relative permittivity [1]. Negative relation between glucose concentration and relative permittivity have also been observed in [172,173,175].

Measurement of the dielectric properties of a material is a function of the resonant or non-resonant response. The resonant response methods are classified primarily as resonator or resonant perturbation types [171,176]. A change in the glucose concentration will cause a change in the dielectric properties of the tissue and thus impedance, and is the input into the sensor circuit. Sensors include microstrip patch antennas [177], spiral microstrip resonator [178,179], open-loop microstrip resonator [180,181,182], split-ring resonators [1,183,184], patch resonators [172] and dielectric resonator antennas [185]. These sensors have different principles of operation, but they are based on the same idea of measuring changes in glucose concentration by measuring the reflected changes in amplitude and resonant frequency of sensors’ response. Generally, these electromagnetic sensors measure the narrowband dielectric properties of tissue based on the resonant frequency. The equivalent circuit model for the sensor is a resonant circuit oscillating at a resonance frequency. The resonant frequency of the sensor can be observed by measuring scattering parameters vs. frequency using a vector network analyzer. The scattering parameters are the reflection coefficient (S11) and the transmission coefficient (S21). The output from the sensor will result in (1) a shift in the resonant frequency of the scattering parameters, (2) a change in the amplitude of the scattering parameters at the resonant frequency, and (3) a change in the 3 dB bandwidths at the resonance frequency, and (4) a change in the quality factor (Q factor) of the sensor. These output measurements reflect changes in the glucose concentration that correlate to the dielectric properties of tissue [186]. The dependency of glucose concentration to the quality factor of an open-loop resonator was recently investigated by measuring the Q factor for different glucose concentration in aqueous solutions [180], blood plasma solutions [181] and on 352 human tongues [182]. The latter study resulted in reliable glucose measurements in some of the human subjects. However, more improvement is necessary to remove the error induced by physiological differences between individuals and different pressure of volunteers’ tongue over the EM sensor. The sensitivity of the EM sensor was further enhanced by design of two electrically coupled open-loop microstrip resonators [187].

The non-resonant response techniques include the free space method, the transmission line method, and the open-ended coaxial probe method [176,188,189,190]. These non-resonant methods use sensors to detect broadband dielectric properties which depend to glucose concentration in tissue [189]. The coaxial line sensor [191], open-ended coaxial line sensor [175], waveguide based sensor [192] and ultra-wideband antennas [193] are used to measure dielectric properties of tissue, and as with the resonant techniques, a VNA is used to measure the tissue scattering parameters as a function of frequency. The scattering parameters can then be converted to dielectric properties of tissue using the software embedded in the VNA or by using conversion methods such as the Nicolson-Ross-Weir method or the NIST iterative conversion method [188,189,194,195]. Once the dielectric property data is collected, it is calibrated to the glucose concentration.

The mmW/MW/UHF sensing method encounters measurement and accuracy challenges, and these are described below, along with possible solutions.

#### 4.4.1. Temperature Fluctuation in Tissue

Tissue dielectric properties are a function of temperature. Jaspard and Nadi measured permittivity and conductivity for temperatures between 25 °C and 45 °C using an impedance-meter and open-ended coaxial line sensor for a frequency range between 1 MHz and 1 GHz [196]. A temperature coefficient was defined as a relative variation in permittivity and conductivity compared to values measured at 25 °C. The results demonstrated that the temperature coefficient for permittivity and conductivity is changed over a wide frequency range. In addition, the sign of the temperature coefficients for permittivity flipped for the frequency range between 1 MHz to 1 GHz. A temperature coefficient of +0.3% °C^−1^ was measured at 1 MHz, and the value of −0.3% °C^−1^ at 1 GHz [196].

The effect of the ambient temperature on the scattering parameter (S21) was investigated by Jang et al., where a complementary split-ring resonator with resonant frequency around 2.9 GHz was developed to measure glucose concentration within a solution of deionized water and glucose [1]. A 0.03 dB variation in amplitude of S21 was observed for a glucose concentration increase from 0 to 400 mg/dL [1]. It was shown that an ambient temperature variation from 20 °C to 40 °C has a more significant effect on the amplitude of S21 compared to the glucose concentration variation from 0 to 400 mg/dL. In this study, a correction function was derived from S21 vs. temperature data and it is applied to remove the effect of temperature from the developed glucose prediction model [1].

To monitor the change in temperature as well as changes in the dielectric properties of tissue, Choi et al. proposed an EM sensor comprising of two spatially separated split-ring resonators [184]. One of the rings is placed close enough to human skin for measuring tissue dielectric, and the second ring is placed a specified distance away from both the human skin and the first ring for measuring tissue temperature. The effect of temperature was eliminated using a temperature correction factor and resulted in a glucose sensor comparable to commercialized sensors.

In order to predict dielectric properties of tissue at frequencies between 0.5 and 20 GHz, and at a temperature between room temperature and 60 °C, a successful technique was proposed in [197] where the dielectric properties of tissue were modeled using a temperature-dependent Cole-Cole and second-order polynomial parameters.

#### 4.4.2. Patient Motion Artifact, Sweat/Humidity, Variation in tissue hydration state, Variation in Hematocrit

Patient motion artifacts and sweating or the humidity level at the tissue surface both interfere with tissue dielectric measurements. The tissue dielectric measurement is also affected by variations in the tissue dehydration state which depends on the humidity in the environment. The effect of dehydration on permittivity and conductivity of tissue was investigated in [198] by performing experiments on freshly excised mouse livers. The dielectric properties of the mice livers were measured using an open-ended coaxial probe for a frequency range between 0.5 and 20 GHz. The results of this study indicated a significant reduction in dielectric properties (permittivity and conductivity) due to tissue dehydration. This highlights the need for controlled experimental conditions when measuring dielectric properties from ex-vivo samples.

The tissue hydration state can be monitored by measuring the NaCl level within the sweat [199]. Eldamak and Fear proposed a patch antenna operating in the range between 2 and 4 GHz to monitor the hydration state of tissue [199]. The proposed sensor measures the hydration state based on changes in the resonant frequency and the amplitude of the reflection coefficient (S11), which are a function of the NaCl level within sweat.

The blood hematocrit level is another factor that interferes with dielectric based glucose measurements. Blood hematocrit refers to the percent volume of red blood cells in the blood, and the dielectric properties of blood depend on the amount of red blood cells. This dependency was investigated by Jaspard et al. by measuring the dielectric properties of animal blood for different levels of hematocrit in the blood (between 20 and 70% volume) for a frequency range between 1 MHz and 1 GHz [200]. Based on this study, an increase in the blood hematocrit level results in a reduction in the blood conductivity over this frequency range. An increase in the blood hematocrit level increases the relative permittivity levels for a frequency range between 1 MHz and 50 MHz, while a reverse trend (decrease in relative permittivity with the increase in hematocrit) occurs for measurements between 50 MHz and 1 GHz. A similar correlation between the blood dielectric properties and the hematocrit level in human blood was reported for a frequency range between 0.2 and 10 MHz [201].

The use of multiple sensors to detect data from perturbing factors is processed in a combined fashion to improve the accuracy of the overall glucose measurement. The concept of using multiple sensors to improve the accuracy of impedance-based continuous glucose monitoring devices is a common practice [202,203,204,205]. Impedance studies by Caduff et al. were investigated by the Pendragon (Zurich, Switzerland) and Biovotion (Zurich, Switzerland) medical device companies. The electrical properties of tissue were measured using an RLC resonant circuit for a frequency range between 1 and 200 MHz [202]. Based on the results of this research, a wrist glucose monitor called Pendra was developed and a post marketing reliability study of the Pendra device was performed for six T1D patients. Results indicated that 4.3% of the readings fell in the unfavorable area (zone E) on the Clarke error grid analysis chart [206]. Caduff’s group continued their work on this technology to improve the sensor [207,208,209]. In recent work by Caduff et al. and Zanon et al., a wearable multi-sensor device worn on the upper arm was developed [203,204]. The device is equipped with an accelerometer, two temperature sensors, one humidity sensor, and diffuse reflectance optical based sensors (to measure hemodynamic changes). In this device, dielectric properties of tissue are measured in three frequency ranges: between 1 kHz and 200 kHz to monitor sweat, between 0.1 and 100 MHz to monitor glucose variation, and between 1 GHz and 2 GHz to monitor subcutaneous water content. Testing the device on 20 T1D patients showed that 86.9 % of glucose readings fell in zone A+B from Clarke error grid analysis, 0.6% in zone C, 12.1% in zone D and 0.4% in zone E [204]. However, further improvements are required in order to eliminate errors associated with daily life routines and errors caused by tissue differences between individuals.

A non-invasive glucose sensor that has shown to provide consistent glucose readings regardless of age, skin type and skin color is the GlucoWise sensor developed by MediWise Ltd. (London, United Kingdom). GlucoWise is currently under development and is based on two technologies: millimeter wave sensing and nano-composite technology [210]. The millimeter wave sensing uses patch antennas with a frequency range between 56 and 61 GHz. Integrating the GlucoWise sensor with the nano-composite films makes the skin temporarily transparent to the wave signal during measurements. Combining the two technologies has been claimed to provide sufficient accuracy for blood glucose monitoring [211].

## 5. Non-Invasive Glucose Sensing Based on Breath Acetone Analysis

Breath analysis is one of the non-invasive approaches for diagnosis and monitoring of diabetes. The human breath contains thousands of volatile organic compounds (VOCs) such as acetone which is found abundantly [212,213]. Acetone is one of the plasma ketone bodies produced in the liver when the acetyl-CoA level increases due to lipolysis, where lipolysis is the body’s response when the glucose supply in the body is insufficient.

People with diabetes have higher levels of acetone compared to healthy people, since insulin acts as an inhibitor for ketones production, and diabetes patients experience low insulin levels. As a result, exhaled breath contains acetone that varies from 300 to 900 ppb in healthy people compared to more than 1800 ppb in individuals with diabetes [42]. Jiang et al. reported that the acetone levels measured in 22 T1D patients, 312 T2D and 52 healthy individuals have the mean values of 4.9 ppm, 1.5 ppm and 1.1 ppm, respectively [44]. Higher levels of breath acetone in diabetes patients imply that breath acetone can be used as a biomarker for a diabetes diagnosis.

The simultaneous single measurement of blood glucose and breath acetone in diabetes patients are reported in the literature [45,214,221,222]. A more comprehensive measurement involving continuous simultaneous acetone and glucose monitoring in order to observe the trends in both measurements are reported in [43,215,216,217,219]. Studies have reported positive correlation, negative correlation and in some cases, no correlation between the two types of measurements, and these studies are summarized in Table 1, Table 2 and Table 3, respectively. Table 1, Table 2 and Table 3 include a summary of blood glucose and breath acetone studies performed on rats and human subjects under various conditions such as fasting, food intake and insulin injection; along with type and frequency of measurements, and breath acetone measurement methods. Glucose infusion occurred in three experiments in which the subjects of the study drank a solution containing 75 g of glucose [217,219,220]. In some clinical studies, animal subjects permit easy control of desired experimental parameters. Although rats share more than 98% DNA with humans, the results from these studies are not necessarily comparable with results obtained from human studies.

The correlation of blood glucose and breath acetone in the literature yields various conclusions (positive, negative and no correlation). The reasons behind this discrepancy are different aspects of their experimental conditions such as insulin injection, glucose injection and food intake. A negative correlation between glucose concentration and breath acetone was observed for T1D subjects under the following conditions during the experiment involving continuous measurements [43,216]:

- No insulin injection
- No glucose ingestion
- No fasting

For these subjects, the glucose levels decrease with time since they are not fed glucose, and the acetone level rises with time because diabetic subjects are susceptible to higher ketones compared to healthy subjects due to insufficient insulin levels. This is expected since insulin acts as an inhibitor for ketoacidosis.

A positive correlation between glucose concentration and breath acetone was observed for T1D patients that were continuously monitored under the following conditions during the experiment [217,218]:

- Insulin injection
- No glucose ingestion
- No fasting

For these patients, as the glucose in the body decreases with passing time, insulin helps the body to use glucose to produce energy. The acetone level also decreases because the patients’ body is not forced to produce ketones for energy (ketones are produced by the body when the glucose in the body is depleted). In this case, there is enough insulin in the body to control ketogenesis, thus preventing ketoacidosis. 

A positive correlation was also observed when 10 healthy human subjects were continuously monitored under the conditions of glucose ingestion and no insulin injection [219]. During the time of the experiment, a rapid increase was observed in the blood glucose level (due to glucose ingestion), and then the glucose level gradually returned to its baseline value. The acetone level had a continuous decreasing trend since the human bodies fed glucose and were not forced to produce ketones for energy.

There are reports of no correlation between glucose concentration and breath acetone during experiments involving single measurements [46,220,221,222,223]. Single measurements do not clearly define the relationship between glucose and breath acetone. There are several other factors (besides glucose levels) that influence the level of breath acetone in the body. These factors may influence the relationships between acetone and blood glucose levels and it is important to consider these effects when designing more controlled and repeatable experiments. Some of the factors that are known to affect breath acetone levels are the following:

- Insulin level
- Biological parameters (human age, human gender)
- Human subjects’ diet
- Intensive exercise (increases breath acetone)
- Alcohol consumption (increases breath acetone)
- Diseases/Illnesses of patients/subjects. (Ex. individuals with epilepsy exhibit higher acetone levels [218])
- Type of diabetes in human subjects (T1D, T2D, healthy) since they may have different metabolic pathways
- The pressure and temperature of the air that human subjects breathe into during breath acetone measurements
- Sampling times (Ex. time after a meal, after fasting, after insulin injection or after glucose consumption) and time of day
- The sampling size should be big enough to generate valid conclusions

The most important challenge in glucose reading through breath acetone measurements is that breath acetone level depends not only on glucose level but also other factors as already mentioned. So, the correlation between breath acetone and blood glucose level is not a simple linear one. A neural network model approach is suited to characterize such a complex system and accurately predict glucose concentrations. The network is trained using different input variables including breath acetone level, insulin level, type of diabetes, nutritional intake, disease, age and gender [224,225]. This needs a comprehensive study in various human subjects with a big sampling size to draw final conclusions.

Another challenge is a possible delay that may exist between changes in blood glucose and the corresponding changes in the breath acetone. Among the 14 studies reviewed in this paper, there is only one study that examines the delay associated with glucose measurements [218]. In this study, the variation of breath acetone level was compared to the blood glucose levels for 3 of the T1D human subjects while using insulin pumps during a 24-h experiment (multiple measurements were taken throughout the 24-h test for each person). A peak in the blood glucose level was observed during the early morning, and the glucose levels fell with time until late afternoon, before dinner. Immediately after dinner, a glucose level peak was observed, followed by a decrease in glucose with time. For one of these patients, the acetone level decreased with a decrease in blood glucose level, and vice versa. In the second patient, a weak correlation between acetone and blood glucose was observed. The measurements associated with the third T1D patient included a 4-h time delay between the breath acetone peaks and the blood glucose peaks [218]. This observation thus suggests more investigation of possible delay between blood glucose and breathe acetone peaks that can differ from one person to another.

The simultaneous continuous monitoring of blood glucose and breath acetone levels is necessary to determine whether a certain delay exists between the two measurements. A key factor for ease of continuous glucose monitoring is the design and implementation of a portable personalized device that measures real time breath acetone levels. Breath acetone levels can be detected using different techniques such as gas chromatography coupled to mass spectrometry (GC-MS), selected ion flow tube mass spectrometry (SIFT-MS), cavity ringdown spectroscopy (CRDS), proton transfer reaction mass spectrometry (PTR-MS), etc. VOC detectors based on these techniques have high selectivity and sensitivity; however, they are not portable. There is an interest to design compact size VOC detectors using nanotechnology. Nanomaterial based gas sensors have been developed to realize real time and portable VOC detector using a semiconducting metal oxide such as titanium dioxide (TiO2) [226], indium loaded tungsten oxide- tin dioxide (WO3-SnO2) [227] and perovskite praseodymium ferrite (PrFeO3) [228].

Recent studies indicate that the measurement of other VOCs within exhaled breath provides additional meaningful information regarding the metabolic pathway. The VOCs are such as methanol, ethanol, methyl nitrate, ethylbenzene, propane, and isoprene that can serve as possible blood glucose biomarkers. For example, Novak et al. demonstrated that methyl nitrate can serve as a biomarker for hyperglycemia in T1D patients [229]. Neupane et al. measured isoprene in the exhaled breath of T1D patients and demonstrated that the level of isoprene increases during hypoglycemia [230]. It was shown that exhaled breath condensate (aerosol) contains 0.01 mM glucose in healthy subjects which correlates with blood glucose levels [231].

The measurement and analysis of more than one VOC can help to yield a more precise estimate of the glucose concentration from these types of measurements. Galassetti et al. improved glucose prediction power by performing multiple linear regressions on both breath ethanol and breath acetone data [219]. In this study, the correlation coefficient between the predicted glucose level and the real value of blood glucose was 0.7.

## 6. Discussion

This review paper presents a multidisciplinary view of non-invasive glucose measurement approaches. Interdisciplinary collaboration is essential for realizing highly accurate non-invasive blood glucose monitoring devices applicable for public use. This paper has been aimed to include not only the engineering and experimental disciplines associated with non-invasive techniques but also the relevant physiological and biochemistry theory related to blood glucose and other tissue components. An effort was made to explain how glucose measurements are affected by physiological factors and how their confounding effect can be minimized.

In-vitro glucose measurements have resulted in acceptable clinical accuracy. These experiments are helping to identify interfering factors and the influence of these factors on the accuracy of glucose sensing measurements. All experimental strategies or theoretical models designed to minimize the effect of many of these interfering factors need to be validated using a multi-layer tissue which provides a realistic physiological setting [232,233]. However, establishing reliability and accuracy during in-vivo experiments is challenging. The functionality and accuracy of all non-invasive systems need to be systematically tested through a variety of standardized experiments on human subjects, to include a variety of conditions such as those described in this paper.

A common challenge for many non-invasive techniques is inter-individual differences in morphology and/or concentration of blood and/or tissue components. These differences can affect the measured signal and consequently the accuracy of glucose readings. The blood and tissue components that influence these measurements include blood proteins (mostly hemoglobin and albumin), red blood cells, fatty tissue, the osmolality of the blood serum, keratin in the epidermis and collagen in the dermis. Selectivity and sensitivity of glucose sensors can be improved by integrating multiple technologies (optical or/and electrical based) into a single platform. Although the various technologies are susceptible to a multitude of interfering factors, the combination of these technologies can compensate for the error associated with any single technology.

Integrating multiple technologies into a single platform affects the complexity of the design, processing algorithms, device size, cost, etc. Thanks to Micro-Electro-Mechanical Systems (MEMS) technology, the multifunctional sensing platforms can be developed with low cost, miniaturized structure and high signal to noise ratio [234]. MEMS devices are mostly made of silicon through microfabrication techniques and have the potential to contribute to the development of a platform integrating multiple technologies (optical or/and electrical based). The output data collected from each technology can be fed to machine learning and/or neural network algorithms in order to develop a glucose prediction model. These algorithms are such as self-organizing map (SOM), feedforward neural network (FNN) and support vector regression [224,225].

Non-invasive techniques that measure glucose within physiological fluids, such as interstitial fluid, salvia and aqueous humor of the eye, are not able to monitor the real-time blood glucose levels due to an unavoidable lag time associated with the change in blood glucose vs. the level of glucose in the physiological fluid. The time needed for the physiological fluid to reflect the blood glucose levels may differ between individuals and further work is required to collect and report the lag times and how they vary for each subject. 

Motion artifacts also contribute to and have an effect on the accuracy of glucose measurements. A motion tracking system and/or a fixed sensor probe on the skin can be used to minimize errors. Other confounding factors are such as temperature and sweating/humidity levels that can be measured using a system with multiple sensors [203,204]. 

In order to counter these effects, multi-technology and multi-sensors can be implemented and tailored to design a non-invasive sensor that is most sensitive to glucose molecules and independent of the many factors that contribute to false readings [65,147,166,167,211]. Artificial intelligence and data analytics is necessary to analyze the data collected and to incorporate it into a prediction model to accurately quantify glucose levels [235].

Table 4 includes the non-invasive glucose measurement devices (based on techniques included in this review) that are currently available or in the development phase. It can be inferred from the methods included in Table 4, that NIR/MIR spectroscopy and tissue dielectric spectroscopy (bio-impedance spectroscopy or mmW/MW/UHF sensing) demonstrate the highest potential for a successful commercial non-invasive glucose sensor. There exist other non-invasive technologies (also described in this paper in Section 3 and Section 4) under development with acceptable accuracy. However, these techniques require additional work to improve repeatability and applicability for a wide range of diverse populations before approval for clinical use is granted.

We have further discussed non-invasive blood glucose sensing approach via tracking change in breath acetone level which is induced by a change in blood glucose concentration. Results obtained by literature regarding the correlation of the breath acetone concentration and blood glucose level are summarized in Table 1, Table 2 and Table 3. Acetone level measurements definitely have the potential to assist with the control of diabetes in patients, however, blood glucose monitoring through measurements of breath acetone needs to be investigated in more controlled circumstances. A generation of reliable algorithms under well documented conditions is needed in order to correlate blood glucose and the breath acetone level in diabetic patients. Single measurements do not clearly define the relationship between blood glucose and acetone levels. Also, continuous monitoring of blood glucose and acetone levels over an extended period of time may clarify and/or confirm if there is a lag between blood glucose and exhaled acetone trends. A more comprehensive study may be one where the breath acetone measurement is correlated to blood glucose levels that are normalized with respect to the level of insulin in the body. A pair of patients with the same glucose levels may have different acetone levels. And different acetone levels can be due to different levels of insulin in the body. By including the level of insulin when reporting blood glucose and acetone levels, a matrix can be developed for patients, medical personnel, and vendors, who are trying to control glucose levels. This acetone vs. glucose data-sheet can be developed as a handout for patients including different tables that show the respected glucose level based on acetone level under different conditions such as insulin level, gender, age, type of diabetes, time of day (fasting or after food intake). However, this type of data requires comprehensive research studies. The algorithm that correlates this type of data (acetone level to blood glucose) needs to account for many conditions, especially the level of insulin level since it significantly impacts the correlation of acetone and blood glucose levels.

## Figures and Tables

**Table 1:** Summary of studies that report a negative correlation between breath acetone and blood glucose concentration.

Ref	Type of Patient, No of Subjects	Instrumentation for Measuring Blood Glucose	Insulin Injection/Infusion Treatment	Control of Food Intake	Type of Meas. (Duration)	Correlation of Breath Acetone with Blood Glucose Concentration	Acetone Meas. Technique
[43]	T1D, 3 rats randomly chosen from 20 rats	Standard Diabetic Management BG meter (Roche, Switzerland)	No history of treatment	No information available during continuous monitoring	Continuous monitoring (6 h)	Negative correlation in each subject (glucose decreases and breath acetone increases)	Cavity Ringdown Spectroscopy
[214]	T1D, 126 Rat Subjects	Blood glucose and ketone monitoring system kit (Optium Xceed, Abbott, USA)	Insulin therapy daily dose of 8 unit/kg (5 days)	4 h of fasting	Single measurement	Weak negative correlation when T1D subjects were ungrouped (Pearson’s R = −0.13); Moderate negative correlation between the mean group acetone and the mean group blood glucose level when T1D rats were grouped into 3 subgroups (Pearson’s R = −0.678)	Cavity Ringdown Spectroscopy
[215]	Non-diabetic, 11 Human Subjects	Using Precision Xtra, electrochemical capillary blood monitor from Abbott, and glucose strips,	No information available	on 1^st^ day, isocaloric meals were given to each subject for breakfast lunch and dinner; on 2^nd^ day, subjects fasted until 7:00 pm (measurements were taken from 10: 00 am to 7:00 pm on this day)	Continuous monitoring (9 Hours); Total of about 55 measurements for all 11 subjects	Negative nonlinear correlation for all subjects (glucose decreases and breath acetone increases); Squared regression coefficient R^2^ = 0.52	Selected Ion Flow Tube-Mass Spectrometry
[216]	T1D, 5 Human Patients	Accu-Chek Active (Roche Diagnostics, Berlin, Germany)	No information available	No information available	Continuous monitoring (7 measurements at different times of day)	Negative correlation in each subject (glucose increases and breath acetone decreases); (R^2^ = 0.92, R^2^ = 0.96, R^2^ = 0.74, R^2^ = 0.45, R^2^ = 0.11)	Commercial acetone gas sensor (TGS 822, 823 Figaro, Arlington Heights, IL, USA Inc)

**Table 2:** Summary of studies that report a positive correlation between breath acetone and blood glucose concentration.

Ref	Type of Patient, No of Subjects	Instrumentation for Measuring Blood Glucose	Insulin Injection/Infusion Treatment	Control of Food Intake	Type of Meas. (Duration)	Correlation of Breath Acetone with Blood Glucose Concentration	Acetone Meas. Technique
[217]	T1D, 8 Human Patients	Intravenous catheter used for blood sampling and hand was warmed to 55C to “arterialize” the venous sample, OGTT performed	Insulin infusion to create hypoglycemia state	Overnight fast	Continuous monitoring (180 min)	Positive correlation in each subject (glucose and acetone decrease) (R^2^ = 0.85, R^2^ = 0.88, R^2^ = 0.90, R^2^ = 0.78, R^2^ = 0.60, R^2^ = 0.86, R^2^ = 0.94, R^2^ = 0.71)	Selected Ion Flow Tube-Mass Spectrometry
[218]	T1D, 30 Human Patients	Standard Self-Management BG meter owned by each patient	Under insulin treatment by wearing an insulin pump	No control	Single measurement per person	Positive correlation between the mean group acetone and the mean group blood glucose level when T1D subjects are grouped by different blood glucose level (R = 0.98, P < 0.02)	Cavity Ringdown Spectroscopy
[218]	T1D, 3 Human Patients	Standard Self-Management BG meter owned by each patient	Under insulin treatment by wearing an insulin pump	Monitoring of food intake during a 24-h test	Continuous monitoring (24 h)	Weak positive correlation in 2 T1D subjects (glucose and acetone peak at food intake, and then glucose and acetone decrease); A 4-h time delay between the breath acetone peaks and the blood glucose peaks in 1 T1D subject	Cavity Ringdown Spectroscopy
[44]	T1D, 20 Human Patients	Standard Diabetic Management BG meter (Roche, Switzerland)	No information available	Measurements were done in 4 different testing conditions: 14 h fast and 2-h post meals (breakfast, lunch and dinner)	Single measurement per person (4 samples taken for each subject under different testing condition)	Weak positive correlation between the mean individual breath acetone and the mean individual blood glucose levels in T1D subjects (R = 0.56, P < 0.005)	Developed breath acetone analyzer based on the Cavity Ringdown Spectroscopy
[43]	T1D, 5 rats	Standard Diabetic Management BG meter (Roche, Switzerland)	Insulin injected for five days, Measurements were done in third and fifth day	No information available during continuous monitoring	Single measurement in the third and fifth day	Weak positive correlation in T1D subjects (Pearson’s R = 0.59, P < 0.05)	Cavity Ringdown Spectroscopy
[45]	T2D, 113 Human Patients	No information available	No information available	8 h of fasting	Single measurement per person	Weak positive correlation (R = 0.32, P = 0.002)	Gas Chromatography/Mass Spectrometry coupled with Solid Phase Micro-Extraction technique
[219]	Non-diabetic, 10 Human Subjects	Intravenous catheter was inserted into basilic vein, OGTT performed	No insulin injection; Serum insulin levels were measured in all subjects and then average values were calculated; A rapid increase in insulin level by 30 min, and peaking at 60 min	Overnight fast	Continuous monitoring (120 min)	Weak positive correlation in each subject; An average individual correlation coefficient of R = 0.4; The average value of acetone had a continuous decreasing trend during experiments, while a rapid increase observed in the average value of glucose which then gradually returned to its baseline value	Gas Chromatography/Mass Spectrometry

**Table 3:** Summary of studies that report no correlation between breath acetone and blood glucose concentration.

Ref	Type of Patient, No of Subjects	Instrumentation for Measuring Blood Glucose	Insulin Injection/Infusion Treatment	Control of Food Intake	Type of Meas. (Duration)	Correlation of Breath Acetone with Blood Glucose Concentration	Acetone Meas. Technique
[44]	T1D, 20 Human Patients; T2D, 312 Human Patients; Non-Diabetic, 52 Human Subjects	Standard Diabetic Management BG meter (Roche, Switzerland)	No information available	Measurements were done in 4 different testing condition: 14 h fast and 2-h post meals (breakfast, lunch and dinner)	Single measurement per person (4 samples taken for each subject under different testing condition)	No clear correlation between the individual breath acetone and the individual blood glucose level in T1D, T2D and healthy subjects	Developed breath acetone analyzer based on the Cavity Ringdown Spectroscopy
[220]	T2D, 22; IGT *, 33; IFG **, 14; RHG ***, 5; Non-Diabetic, 67 Human Subjects	No information available	No information available	10-h fasting (no eating during the experiment)	Single measurement every hour for 2.5 h (0 h (initial measurement), 1 h, 2 h) in all group	No clear correlation at any time (0 h,1 h,2 h) between individual breath acetone and individual blood glucose level in all groups	Ion-molecule-Reaction Mass Spectrometer (V&F Analysen and Messtechnik GmbH, Austria)
[46]	T2D, 149 Human Patients; Non-diabetic, 42 Human Subjects	Standard Diabetic Management BG meter (Roche, Switzerland)	No information available	Measurements were done in 4 different testing condition: 14 h fast and 2-h post meals (breakfast, lunch and dinner)	Single measurement per person (4 samples taken for each subject under different testing condition)	No clear correlation at any condition between individual breath acetone and individual blood glucose level in T2D and healthy subjects	Cavity Ringdown Spectroscopy
[221]	T1D, 3 Human Patients (2 minors and 1 adult)	Glucose meter (Bayer Contour Link)	Insulin Dispensers (2 of subjects who were minors) and manual insulin injection (1 Adult)	Overnight fast	Single measurement per person	No clear correlation in T1D subjects	Quantum Cascade Laser Spectroscopy
[222]	T2D, 38 Human Patients	Abbot Optium Xceed	Various Treatment: Diabetic diet(6), Metformin monotherapy(21), Insulin plus metformin(5) Combinations of oral therapy(5)	No fasting and no eating one hour before the test	Single measurement per person	No correlation in T2D subjects	Selected Ion Flow Tube-Mass Spectrometry
[223]	T1D, 20 Human Patients	Standard Diabetes Monitoring Meter (Abbott Diabetes Care Ltd., UK, FreeStyle Optium)	No information available	Minimum 8 h overnight fast	Single measurement once per day for 30 days	No clear correlation between the mean individual blood glucose and the mean individual breath acetone in T1D subjects (R = 0.17, P = 0.43)	Developed breath acetone analyzer based on the Cavity Ringdown Spectroscopy

* Impaired glucose tolerance, ** Impaired fasting glycaemia, *** Reactive hypoglycemia.

**Table 4:** Summary of fully non-invasive glucose sensors commercially available or under development based on the techniques reviewed in this paper.

Non-invasive Device	Technology	Company	Meas. Area	Description	Ref
TensorTip Combo Glucometer	VIS-NIR spectroscopy	Cnoga Medical Ltd. (Israel)	Fingertip	State: approved for use in numerous countries worldwide; Comprised of four LEDs (600 nm–1000 nm) and a camera sensor; Subjects enrolled: 14 healthy, 6 T1D and 16 T2D; Accuracy based on consensus error grid analysis: 96.6% in zone A and 3.4% in zone B.	[64,65,66]
Wizmi	NIR spectroscopy	Wear2b Ltd. (Israel)	Wrist	State: Proof of concept acquisition; Subjects enrolled: 32 healthy women; Accuracy based on Clarke error grid analysis: 93% in zone A, 7% in zone B.	[236]
LTT device developed by the research group of Quantum Science and Technology	MIR spectroscopy	Light Touch Technology Ltd. (Japan)	Finger	State: Under development; High brightness light source in MIR range (6000–9000 nm) was developed using optical parametric oscillator technology of solid-state laser; Accuracy results: not available.	[237,238]
Biovotion	Bioimpedance Spectroscopy	Biovotion Ltd. (Switzerland)	Arm	State: proof of concept acquisition; Is a multi-sensor system; Measuring dielectric properties of tissue at three different frequency region: (1–200 kHz), (0.1–100 MHz) and (1–2 GHz); Subjects enrolled: 20 T1Ds; Accuracy based on Clarke error grid analysis: 86.9% in A + B, 0.6% in C, 12.1% in D, 0.4% in E.	[203,204,205]
GlucoWise	Millimeter wave spectroscopy, nanocomposite technology	MediWise Ltd. (UK)	Between the thumb and forefinger	State: under development; Millimeter wave transmission measurement at a range between 56 GHz and 61 GHz using microstrip patch antennas; Accuracy results: not available.	[210,211]
